# Dopamine D_2_ receptor modulates Wnt expression and control of cell proliferation

**DOI:** 10.1038/s41598-019-52528-4

**Published:** 2019-11-14

**Authors:** Fei Han, Prasad Konkalmatt, Chaitanya Mokashi, Megha Kumar, Yanrong Zhang, Allen Ko, Zachary J. Farino, Laureano D. Asico, Gaosi Xu, John Gildea, Xiaoxu Zheng, Robin A. Felder, Robin E. C. Lee, Pedro A. Jose, Zachary Freyberg, Ines Armando

**Affiliations:** 1 Department of Medicine, School of Medicine and Health Sciences, The George Washington University, Washington, DC 20052 USA; 2 Department of Computational & Systems Biology, University of Pittsburgh, Pittsburgh, PA 15213 USA; 3 Institute of Human Nutrition, College of Physicians & Surgeons, Columbia University, New York, NY 10032 USA; 4 Department of Psychiatry, University of Pittsburgh, Pittsburgh, PA 15213 USA; 5 Department of Pathology, The University of Virginia, Charlottesville, VA 22904 USA; 6 Department of Pharmacology and Physiology, School of Medicine and Health Sciences, The George Washington University, Washington, DC 20052 USA; 7 Department of Cell Biology, University of Pittsburgh, Pittsburgh, PA 15213 USA; 8 Present Address: Kidney Disease Center, First Affiliated Hospital, College of Medicine, Zhejiang University, Hangzhou, China

**Keywords:** Transcriptional regulatory elements, Acute kidney injury

## Abstract

The Wnt/β-catenin pathway is one of the most conserved signaling pathways across species with essential roles in development, cell proliferation, and disease. Wnt signaling occurs at the protein level and via β-catenin-mediated transcription of target genes. However, little is known about the underlying mechanisms regulating the expression of the key Wnt ligand Wnt3a or the modulation of its activity. Here, we provide evidence that there is significant cross-talk between the dopamine D_2_ receptor (D2R) and Wnt/β-catenin signaling pathways. Our data suggest that D2R-dependent cross-talk modulates Wnt3a expression via an evolutionarily-conserved TCF/LEF site within the *WNT3A* promoter. Moreover, D2R signaling also modulates cell proliferation and modifies the pathology in a renal ischemia/reperfusion-injury disease model, via its effects on Wnt/β-catenin signaling. Together, our results suggest that D2R is a transcriptional modulator of Wnt/β-catenin signal transduction with broad implications for health and development of new therapeutics.

## Introduction

Dopamine (DA) signaling, although traditionally studied in the central nervous system, is increasingly implicated in regulating the function of peripheral organs, including the pancreas and kidney^1–5^. Renal proximal tubule cells express components of the DA biosynthetic machinery as well as both D_1_-like (D_1_, D_5_) and D_2_-like (D_2_, D_3_, D_4_) DA receptors^5–7^. These cells therefore endogenously synthesize DA from circulating precursors, and utilize the newly synthesized DA locally, independent of the nervous system, to signal in an autocrine/paracrine manner^6,8,9^. This renal DA signaling regulates sodium transport to maintain fluid and electrolyte homeostasis and normal blood pressure^7–10^. Knockdown of any of the five DA receptor subtypes consequently results in increased blood pressure in mice^4,10,11^. Moreover, kidney-specific downregulation of the DA D_2_ receptor (D2R) increases oxidative stress and causes renal tubulointerstitial fibrosis and glomerulosclerosis^11–14^. Nevertheless, the precise mechanisms of D2R signaling and their relationships to renal physiology and disease remain poorly understood.

D_2_-like DA receptors, including D2R, are class A G protein-coupled receptors (GPCRs) that signal via two downstream intracellular pathways: **(i)** G protein-dependent, arrestin-independent, and **(ii)** G protein-independent, arrestin-dependent pathways^15–17^. In the G protein-dependent pathway, D2R activation results in the recruitment of Gα_i/o_ and ultimately leads to decreased levels of cyclic AMP (cAMP) synthesis^16,18^. In the G protein-independent, arrestin-dependent pathway, recruitment of arrestin 3 (β-arrestin-2) causes D2R internalization and inactivation of additional extracellular D2R signaling^19^. This β-arrestin-2-dependent pathway also leads to the recruitment of protein phosphatase 2 A (PP2A) and serine/threonine kinase AKT to the β-arrestin-2/D2R complex^17,19,20^. PP2A dephosphorylates AKT, leading to inactivation of AKT^21^. AKT regulates another key signaling kinase, glycogen synthase kinase β (GSK3β), through phosphorylation. In its non-phosphorylated state, GSK3β is constitutively active, whereas AKT-induced phosphorylation inactivates GSK3β^20^. This D2R and β-arrestin-2-dependent AKT regulation of GSK3β has been increasingly implicated in neuronal physiology and pathophysiology, as well as in the mechanisms of both antipsychotic drugs and lithium action^18,22–24^. Notably, while D2R signals via G-protein-dependent and -independent pathways in response to stimulation, evidence also suggests that there may be an additional component of constitutive activity in both pathways^25–27^.

In addition to AKT’s roles in β-arrestin-2-dependent D2R signaling, this kinase may also contribute to the regulation of signaling within the Wnt/β-catenin pathway^15,28,29^. The Wnt/β-catenin pathway is nearly ubiquitous across species and plays numerous critical roles in development and in both cell physiology and pathology including cancer^30^. This signaling pathway is comprised of Frizzled (FZD) Wnt receptors and the LRP5/6 co-receptor in association with Dishevelled, a scaffolding protein that activates a β-catenin destruction complex that includes Axin, Adenomatous polyposis coli (APC), and GSK3β^28,31,32^. In the absence of a stimulus, β-catenin, a transcriptional co-activator of Wnt target genes, is phosphorylated by active GSK3β and directed to the proteosomal pathway for degradation^31^. However, following activation of FZD/LRP5/6 by ligands, including Wnt3a, β-catenin translocates into the nucleus and binds to T-cell factor (TCF)/lymphoid enhancer-binding factor (LEF) proteins to stimulate Wnt target gene transcription^30,31,33,34^. Significantly, the ability of β-catenin to evade proteosomal destruction and initiate gene transcription is mediated by inactivation of GSK3β activity. GSK3β activity may be inhibited through phosphorylation of serine 9 or 21 through the actions of several kinases including AKT, protein kinase A (PKA), and integrin-linked kinase (ILK)^32,35,36^. Additionally, GSK3β activity is inhibited in response to treatment with growth factors including insulin/insulin-like growth factor I (IGF-I), nerve growth factor (NGF), or through inhibitory auto-phosphorylation^32,37–42^. Here, given AKT’s prominent role in D2R′s G protein-independent, arrestin-dependent pathway, we focus on D2R-mediated GSK3β inactivation via AKT phosphorylation^15,43^ and the resulting effects on β-catenin-driven changes in gene expression.

Since AKT plays important roles in both D2R and Wnt/β-catenin signaling pathways, we asked whether there is an AKT-dependent crosstalk between these two pathways where effects on AKT activity in one pathway may modulate downstream effects in the other. Moreover, we examined whether stimulation or inactivation of D2R signaling modulates β-catenin-mediated effects on transcription of Wnt pathway target genes. We established both *in vitro* cellular and *in vivo* animal models in both human and mouse renal proximal tubule cells to elucidate D2R′s role in modulating the Wnt/β-catenin signaling pathway, given the importance of both D2R and Wnt signaling pathways in this cell type to kidney function including blood pressure regulation^6,11,12^. Using these models, we demonstrate a new paradigm by which stimulation of a GPCR, D2R, modulates Wnt/β-catenin signaling, Wnt3a expression, and cell proliferation in healthy and disease states, via its effects on gene transcription.

## Results

### β-arrestin-2-dependent AKT and GSK3β activities are modulated by D2R in renal proximal tubule cells

We examined dopaminergic, G protein-independent signaling in renal proximal tubule cells, since, in mice and humans, these cells endogenously express D2R^7,13,14^, as well as key proteins in the β-arrestin-2-dependent pathway including GSK3β, AKT, and PP2A^44–46^. However, to date, the extent of endogenous renal expression of β-arrestin-2 and its conservation across species remain unclear. We found that β-arrestin-2 was endogenously expressed in mouse renal cortex, as well as in both mouse and human renal proximal tubule cells (Supplementary Fig. S1). Interestingly, comparison of β-arrestin-2 expression in human renal proximal tubule cells relative to Gapdh closely resembled β-arrestin-2 expression in mouse renal cortex (Supplementary Fig. S1).

**Figure 1:**
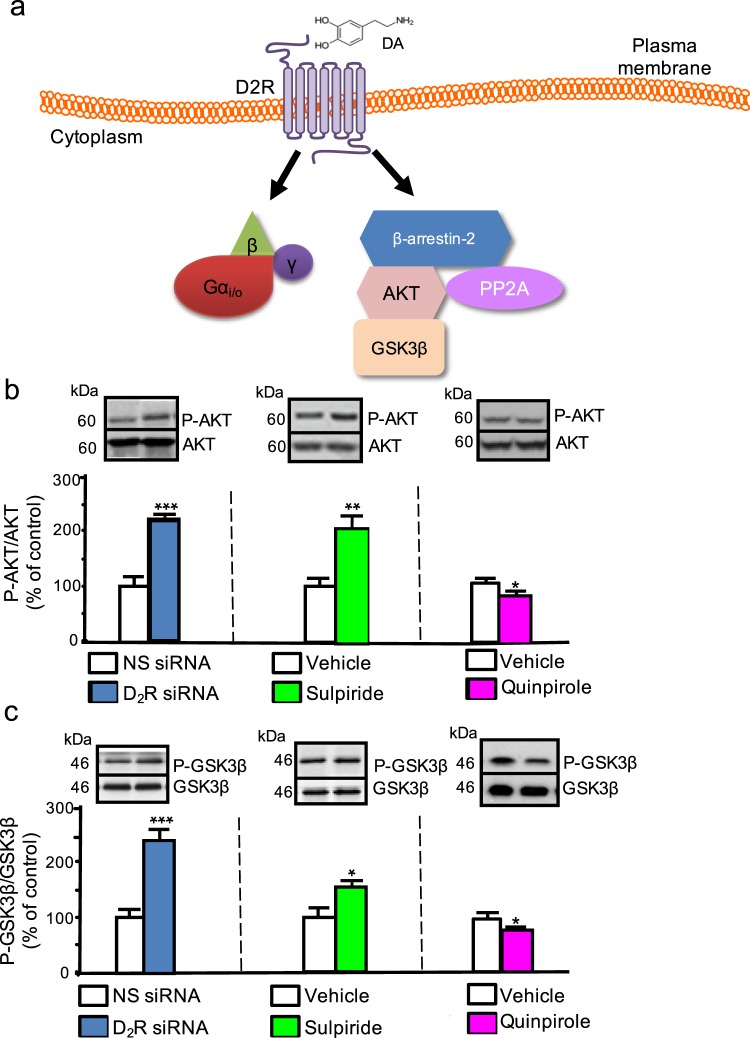
AKT and GSK3β phosphorylation is modulated by D2R. (**a**) Model of D2R modulation of AKT/GSK3β signaling. Binding of dopamine (DA) to the DA D_2_ receptor (D2R) recruits β-arrestin-2, a scaffolding protein, along with the kinase AKT and the phosphatase PP2A to the receptor independently of Gα_i/o_ signaling. PP2A dephosphorylates AKT, inactivating the kinase. Phospho-AKT (P-AKT) is responsible for phosphorylating constitutively active GSK-3β, inactivating it. Thus, D2R-mediated AKT inactivation ultimately increases levels of active, non-phosphorylated GSK-3β. **(b)** D2R knockdown in mouse renal proximal tubule cells (mRPTCs) via D2R siRNA (72 hr) caused a 130% increase in AKT phosphorylation at the catalytic/stimulatory T308 site, relative to the non-silencing (NS) siRNA control. Acute treatment with D2R antagonist sulpiride (1 µM, 6 hr) doubled AKT phosphorylation, relative to the vehicle control. D2R agonist quinpirole (1 µM, 24 hr) reduced AKT phosphorylation by 30% compared with the vehicle control. **(c)** D2R knockdown by D2R siRNA in mRPTCs caused a 150% increase in GSK3β phosphorylation (P-GSK3β) at the inhibitory S9 site, while acute sulpiride treatment also increased GSK3β phosphorylation by 50% compared with the respective controls. D2R activation by quinpirole decreased GSK3β phosphorylation by 20%, compared with vehicle. Results are represented as the ratio of P-AKT (T308)/total Akt, and P-GSK3β (S9)/total GSK3β and then normalized as percentage of NS siRNA or vehicle (% of control). All results are represented as the mean ± SEM conducted on n ≥ 3 separate experimental dates and performed in triplicate. *P < 0.05, ***P < 0.001; n = 3–4/group; Student’s t-test.

We determined if mouse renal cortex, as well as mouse and human renal proximal tubule cells, can serve as novel experimental systems to further probe the β-arrestin-2-dependent arm of D2R signaling. Specifically, we explored the following signaling model: (**1**) D2R activation leads to dephosphorylation of active, phosphorylated AKT (P-AKT) and, (**2**) in the setting of decreased P-AKT, repressive phosphorylation of GSK3β is also reduced, thereby increasing GSK3β kinase activity (Fig. 1a). Consistent with this model, siRNA-induced D2R knockdown increased levels of P-AKT at the catalytic/stimulatory T308 phosphorylation site^47,48^ in mouse renal proximal tubule cells (Fig. 1b; original blots shown in Supplementary Fig. S2). We confirmed that these changes were due to effective D2R siRNA-mediated knockdown of D2R protein levels (Supplementary Fig. S3). To control for potential long-term adaptation to D2R downregulation, we also examined the effects of acute D2R blockade using sulpiride, an established D2R antagonist. Acute sulpiride treatment also increased P-AKT T308 levels similar to that found in the siRNA-mediated D2R knockdown (Fig. 1b). Conversely, treatment with the D2R agonist quinpirole decreased P-AKT T308 levels in these cells (Fig. 1b). Based on these data and the above model, we asked whether D2R-dependent changes in AKT phosphorylation produce corresponding alterations in GSK3β phosphorylation. siRNA-induced D2R knockdown increased levels of inactive phospho-GSK3β [P-GSK3β at the inhibitory S9 position^40^] (Fig. 1c, Supplementary Fig. S2); acute sulpiride treatment similarly elevated P-GSK3β levels (Fig. 1c). By contrast, acute treatment with D2R agonist quinpirole decreased P-GSK3β levels (Fig. 1c). We further validated our model in human renal proximal tubule cells. As in mouse renal proximal tubule cells, we found that either siRNA-mediated D2R knockdown or D2R antagonism by sulpiride increased phosphorylation of both AKT and GSK3β, while D2R stimulation by quinpirole decreased the phosphorylation of these kinases (Supplementary Fig. S4). Our data therefore suggest that these mechanisms are conserved across species.

### D2R modulates β-catenin phosphorylation and transcriptional activity in the kidney and pancreas

**Figure 2:**
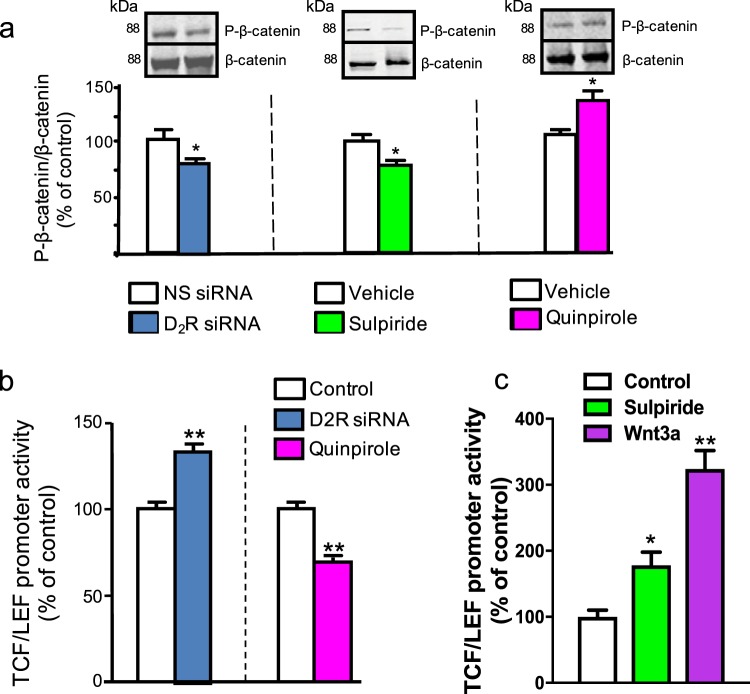
D2R modulates β-catenin phosphorylation and TCF/LEF activity. (**a**) siRNA knockdown of D2R (D2R siRNA) in mouse renal proximal tubule cells (mRPTCs) caused a 20% decrease in phosphorylated β-catenin (P-β-catenin, left panel) compared with the non-silencing (NS) siRNA control (72 hr). Acute sulpiride (D2R antagonist) treatment (1 µM, 6 hr) similarly reduced β-catenin phosphorylation by 25% compared with the vehicle control. Conversely, D2R agonism by quinpirole (1 µM, 24 hr) increased β-catenin phosphorylation, relative to the vehicle control (right panel). Results are represented as the ratio of P-β-catenin/total β-catenin, and then normalized as the percentage of NS siRNA or vehicle (% of control). **(b)** β-catenin-mediated TCF/LEF luciferase reporter activity was significantly increased by 40% via siRNA-mediated D2R knockdown and decreased by 36% via 1 µM quinpirole treatment to stimulate D2R activity; results are normalized to the respective controls. **(c)** Acute sulpiride treatment significantly increased TCF/LEF promoter activity by 80%, while direct stimulation by Wnt3a (100 ng/ml, 2 hr) more than doubled (224%) TCF/LEF promoter activity, relative to vehicle control. All data are represented as the mean ± SEM conducted on n ≥ 3 separate experimental dates and performed in triplicate. *P < 0.05, **P < 0.01; n = 4–5/group, Student’s t-test for (**a**,**b**); one-way ANOVA and Holm-Sidak post-hoc test for (**c**).

In addition to its role as a mediator of D2R signaling, GSK3β is a major regulator of gene transcription via its phosphorylation of the nuclear transcriptional co-activator, β-catenin^49,50^. Phosphorylation of β-catenin by GSK3β increases β-catenin’s rate of proteasome-dependent degradation, thereby reducing β-catenin-dependent transcription of target genes^15,31^. Consistent with our findings that siRNA-mediated D2R knockdown decreased levels of active GSK3β (Fig. 1c), we also observed lower levels of phosphorylated β-catenin (P-β-catenin) in mouse renal proximal tubule cells when D2R expression was either knocked down via D2R siRNA or acutely blocked by sulpiride treatment (Fig. 2a). By contrast, D2R activation by quinpirole increased β-catenin phosphorylation in these cells (Fig. 2a). As with AKT and GSK3β, we replicated D2R-mediated effects on β-catenin phosphorylation in human renal proximal tubule cells (Supplementary Fig. S4).

On a functional level, we examined whether D2R′s effects on β-catenin phosphorylation modulate β-catenin-initiated transcription at TCF/LEF promoter sites. Using a luciferase-based TCF/LEF transcriptional activity assay, we found that decreased D2R signaling either through siRNA-induced D2R knockdown or acute sulpiride treatment elevated TCF/LEF reporter activity (Fig. 2b,c). By contrast, D2R activation by quinpirole decreased TCF/LEF transcriptional activity (Fig. 2b). Notably, while acute D2R blockade by sulpiride has a stronger stimulatory effect on TCF/LEF transcriptional activity relative to D2R siRNA knockdown, these D2R-mediated effects are weaker than direct Wnt3a stimulation (Fig. 2b,c). Overall, our data suggest that D2R mediates changes in transcription through its effects on β-catenin phosphorylation in renal proximal tubule cells.

We also examined whether D2R effects on β-catenin-mediated TCF/LEF transcriptional activity were generalizable to other cell types and tissues beyond the kidney. We therefore determined whether D2R could modulate TCF/LEF transcriptional activity in INS-1E cells, an insulin-secreting rat pancreatic β-cell-derived cell line^51^. We chose this cell line since DA and D2R-dependent signaling may play important roles in β-cell physiology, serving as regulators of glucose-stimulated insulin secretion (GSIS)^1,52–54^. We used the D2R agonist bromocriptine since bromocriptine is a highly potent and effective modulator of GSIS in INS-1E cells^55,56^. Consistent with our data in the kidney, we found that overnight treatment of INS-1E cells with bromocriptine significantly inhibited TCF/LEF transcription (Supplementary Fig. S5). These data suggest that D2R′s putative role as a negative regulator of β-catenin-mediated TCF/LEF transcriptional activity is conserved across cell types and species.

### *DRD2* single nucleotide polymorphisms (SNPs) decrease β-catenin phosphorylation in human renal proximal tubule cells

**Figure 3:**
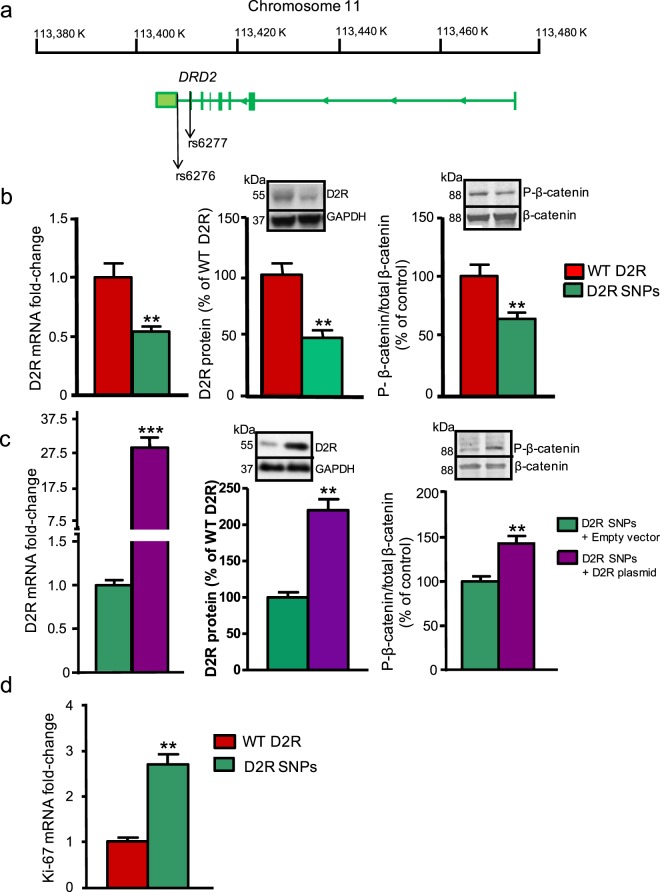
*DRD2* SNP cluster rs6276, rs6277 decreases D2R expression, modulates β-catenin phosphorylation and increases cell proliferation *in vitro*. (**a**) Schema of the relative positions of the SNPs rs6276 and rs6277 in the human *DRD2* gene. **(b)** The joint presence of SNPs rs6276 and 6277 in human renal proximal tubule cells reduced D2R mRNA levels by 50% (left panel) along with a concomitant 50% decrease in D2R protein levels (middle panel). Levels of phospho-β**-**catenin (P-β**-**catenin) also decreased by 40%, relative to control cells without these SNPs (right panel). Insets show representative immunoblots. **(c)** Transfection of the *DRD2* SNP-containing cells with a human *DRD2* plasmid without the SNPs significantly boosted D2R mRNA levels 30-fold (left panel), as well as D2R protein levels 2.2-fold (middle panel). Increasing wild-type D2R expression in these cells also increased P-β**-**catenin levels (right panel, inset showing western blot), relative to the empty vector control. **(d)** Presence of *DRD2* SNPs rs6276 and rs6277 led to a 2.7-fold increase in cellular proliferation as indicated by the Ki-67 marker. All data are represented as the mean ± SEM conducted on n ≥ 3 separate experimental dates and performed in triplicate. **P < 0.01, ***P < 0.001; n = 3–4/group; Student’s t-test.

The *DRD2* gene encoding D2R is highly polymorphic. Several *DRD2* SNPs modify levels of D2R expression, including those expressed in human renal proximal tubule cells^13^. We focused on the combination of two common SNPs in the human *DRD2* gene, rs6276 and rs6277 (Fig. 3a), previously shown to increase vulnerability to renal inflammation independently of blood pressure^13^. We found that the presence of these two SNPs significantly decreased both mRNA and protein expressions of D2R in human renal proximal tubule cells relative to control cells without the SNPs (Fig. 3b). Consistent with our D2R siRNA data in mouse proximal renal tubule cells (Fig. 2a), the presence of these *DRD2* SNPs also diminished the levels of P-β-catenin (Fig. 3b). Conversely, increasing levels of D2R expression in the SNP background via heterologous D2R overexpression led to a concomitant increase in P-β-catenin (Fig. 3c).

### D2R modulates cell proliferation *in vitro* and *in vivo* via Wnt3a

There is a well-known relationship between levels of β-catenin and its effects on cellular proliferation. As levels of non-phosphorylated β-catenin rise, the resulting elevation in β-catenin-driven transcription increases cell proliferation^57–59^. Consistent with these reports, we found that Ki-67, a marker of cell proliferation^60^, was increased 2.7-fold in human renal proximal tubule cells containing rs6276 and rs6277 SNPs (Fig. 3d). Similarly, siRNA-induced D2R knockdown also increased cell proliferation in mouse renal proximal tubule cells (Supplementary Fig. S6). These data suggest a relationship not only between D2R and β-catenin phosphorylation, but also between D2R and cell proliferation.

**Figure 4:**
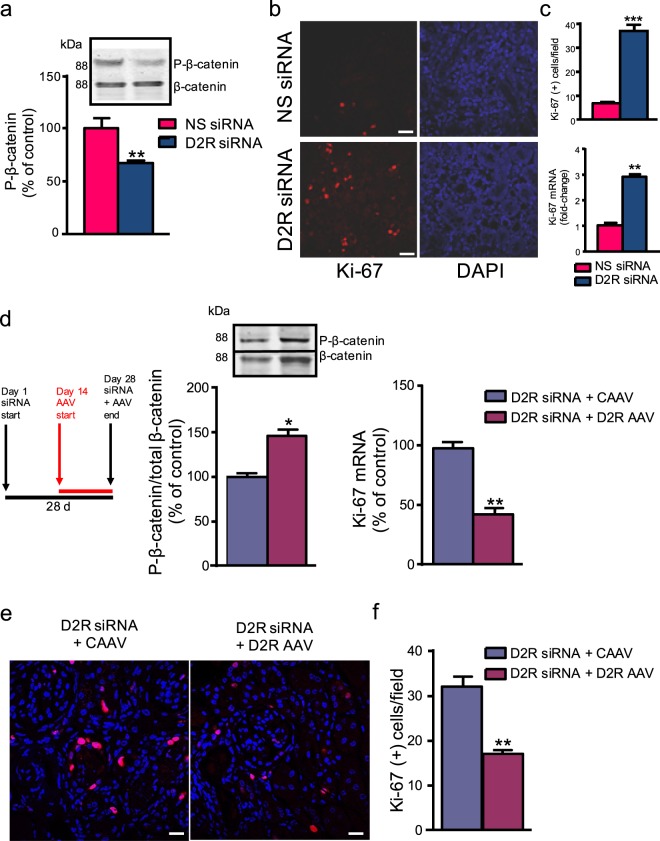
D2R expression modulates renal cell proliferation *in vivo*. (**a**) Kidney-selective D2R knockdown *in vivo* via siRNA (D2R siRNA) decreased β-catenin phosphorylation (P-β-catenin) in mouse renal cortex by 40% relative to the NS siRNA control. **(b)** D2R siRNA knockdown also increased the expression of the cell proliferation marker Ki-67. Merged confocal images show Ki-67 (red) and DAPI nuclear staining (blue); magenta shows colocalization. Scale bar = 50 µm. **(c)** Quantification of the Ki-67-positive cells in the renal cortex revealed significant increases in the number of Ki-67-positive cells/field along with a 3-fold increase in Ki-67 mRNA following D2R siRNA treatment compared with control mice treated with the NS siRNA control; n ≥ 4 for all groups. **(d)** Rescue of D2R expression via AAV-mediated renal D2R overexpression (D2R AAV) reversed the effects of *in vivo* D2R siRNA on β-catenin phosphorylation by raising P-β-catenin levels (left panel); inset shows a representative immunoblot. Alongside is the schedule for the respective siRNA and AAV treatments. Ki-67 expression was decreased in the D2R AAV-treated kidneys by 50% in comparison with mice treated with control AAV (CAAV, right panel). **(e)** Confocal immunofluorescence imaging of the D2R AAV rescue of D2R siRNA knockdown showed a 2-fold decrease in Ki-67-positive cells. Merged confocal images show DAPI (blue) and Ki-67 (red); magenta shows colocalization. Scale bar = 50 µm. **(f)** Accompanying quantification of Ki-67-positive cells in response to D2R AAV rescue of D2R siRNA knockdown. All data are represented as the mean ± SEM. *P < 0.05, **P < 0.01; n = 3–4/group; Student’s t-test.

Building upon our *in vitro* cellular data, we developed an *in vivo* model to further study the relationship between D2R and cell proliferation. *In vivo* renal-selective D2R knockdown by the renal subcapsular infusion of D2R siRNA significantly decreased D2R expression (Supplementary Fig. S7A), and diminished levels of P-β-catenin (Fig. 4a). Consistent with our earlier *in vitro* observations in *DRD2* SNP-containing cells (Fig. 3), we observed an increase in the number of Ki-67-positive cells in renal sections (Fig. 4b,c). D2R knockdown was also associated with increased Ki-67 expression at the mRNA level in renal cortex (Fig. 4c). By contrast, *in vivo* rescue of D2R expression via retrograde ureteral infusion of D2R adeno-associated virus (AAV) increased D2R expression 10-fold (Supplementary Fig. S7b) and reversed the effects of renal-selective D2R knockdown on β-catenin phosphorylation and Ki-67 mRNA and protein expressions (Fig. 4d–f).

**Figure 5:**
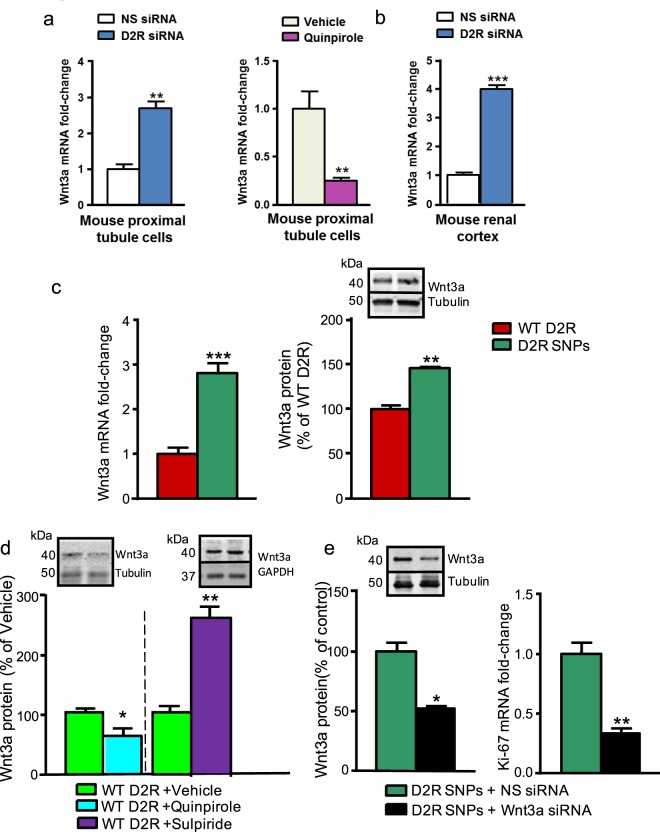
D2R modulates Wnt3a expression. (**a**) D2R siRNA (72 hr) increased Wnt3a mRNA levels 2.6-fold (left panel) in mouse renal proximal tubule cells. Conversely, the D2R agonist quinpirole (1 µM, 24 hr) decreased Wnt3a expression by 70% in these cells (right panel). (**b**) Kidney-selective D2R knockdown *in vivo* via renal subcapsular D2R siRNA infusion similarly increased Wnt3a expression 4-fold in mouse renal cortex (left panel). (**c**) Human renal proximal tubule cells with D2R expression-reducing SNPs rs6276 and rs6277 had increased Wnt3a mRNA levels (2.9-fold, left panel) and protein expression (1.5-fold, right panel) in the setting of diminished D2R expression, relative to cells without these SNPs (wild-type, WT). **(d)** D2R agonist quinpirole (1 µM, 24 hr) decreased Wnt3a protein by 44% in the SNP-free wild-type (WT) human renal proximal tubule cells compared with vehicle. Acute treatment with D2R antagonist sulpiride (1 µM, 6 hr) significantly increased Wnt3a protein by 180% in the WT human renal proximal tubule cells compared with vehicle. **(e)** Wnt3a knockdown via siRNA (Wnt3a siRNA) significantly decreased Wnt3a protein levels (right panel) in human renal proximal tubule cells expressing SNP cluster rs6276 and 6277 and partially attenuated the increased levels of proliferation in these cells, as indicated by the decrease in Ki-67 mRNA (right panel). All data are represented as the mean ± SEM. For Panels (a,c–e): all cell experiments were conducted on n ≥ 3 separate experimental dates and performed in triplicate. For Panel (b): all animal experiments used n = 4–5 animals per group; measurements were performed in triplicate. *P < 0.05, **P < 0.01, ***P < 0.001; Student’s t-test.

Since Wnt3a is a powerful mediator of physiological and pathological cell proliferation via signaling through β-catenin^61–63^, we determined whether D2R′s effects on cell proliferation are modulated through Wnt3a. We found that *in vitro* D2R siRNA knockdown increased Wnt3a expression 2.5-fold, while D2R activation with quinpirole decreased Wnt3a expression by 80% in mouse renal proximal tubule cells (Fig. 5a). *In vivo* knockdown of D2R by the renal subcapsular infusion of D2R siRNA increased Wnt3a expression to an even greater degree (4-fold increase; Fig. 5b). Moreover, in human renal proximal tubule cells with the *DRD2* rs6276 and rs6277 SNPs, the decreased D2R expression was associated with increased Wnt3a mRNA and protein levels (Fig. 5c). Similarly, acute D2R antagonism by sulpiride significantly elevated Wnt3a protein expression in wild-type (non-SNPs) human renal proximal tubule cells, controlling for potential confounding compensatory effects due to lower levels of D2R expression (Fig. 5d). By contrast, treatment of the wild-type (non-SNPs) human renal proximal tubule cells with D2R agonist quinpirole reduced Wnt3a protein levels (Fig. 5d). Although our data strongly suggested links between D2R-mediated changes in both Ki-67 and Wnt3a expression, we sought to determine whether these D2R effects on proliferation are mediated via Wnt3a. Consequently, we knocked down Wnt3a via siRNA in the human renal proximal tubule cells with D2R SNPs and, along with the expected decreases in Wnt3a protein, we also observed significantly decreased expression of Ki-67 (Fig. 5e). To examine the effects of D2R signaling on Wnt3a expression *in vivo*, we knocked down the renal expression of D2R *in vivo* via renal subcapsular injection of D2R siRNA. We found an increase in the mRNA expression of Wnt3a, consistent with our model. Renal-selective rescue of D2R function in the siRNA-injected mice by concurrent AAV overexpression of D2R reduced this Wnt3a increase (Supplementary Fig. S8). Nevertheless, because siRNA-induced D2R knockdown and overexpression were sequential, it is possible that remaining increases in Wnt3a expression caused by the original D2R knockdown led to signaling changes that affected Wnt3a expression that subsequent D2R overexpression was unable to override.

We determined whether D2R modulation of Wnt3a signaling occurred in cell types outside the kidney by again examining pancreatic β cell-derived INS-1E cells. We found that INS-1E cells responded to Wnt3a in a dose-dependent manner by progressively increasing TCF/LEF-mediated transcription (Supplementary Fig. S9a). Furthermore, D2R stimulation by agonist bromocriptine significantly reduced the Wnt3a-induced increase in TCF/LEF signaling (Supplementary Fig. S9b). We confirmed these findings, showing that D2R stimulation with additional agonists including DA or quinpirole also significantly diminished the Wnt3a-induced increase in TCF/LEF activity (Supplementary Fig. S9b). These data suggest that the links between D2R and Wnt3a signaling and the resulting changes in gene transcription are conserved across mammalian cell types.

### Wnt3a expression is regulated by β-catenin and TCF/LEF signaling

**Figure 6:**
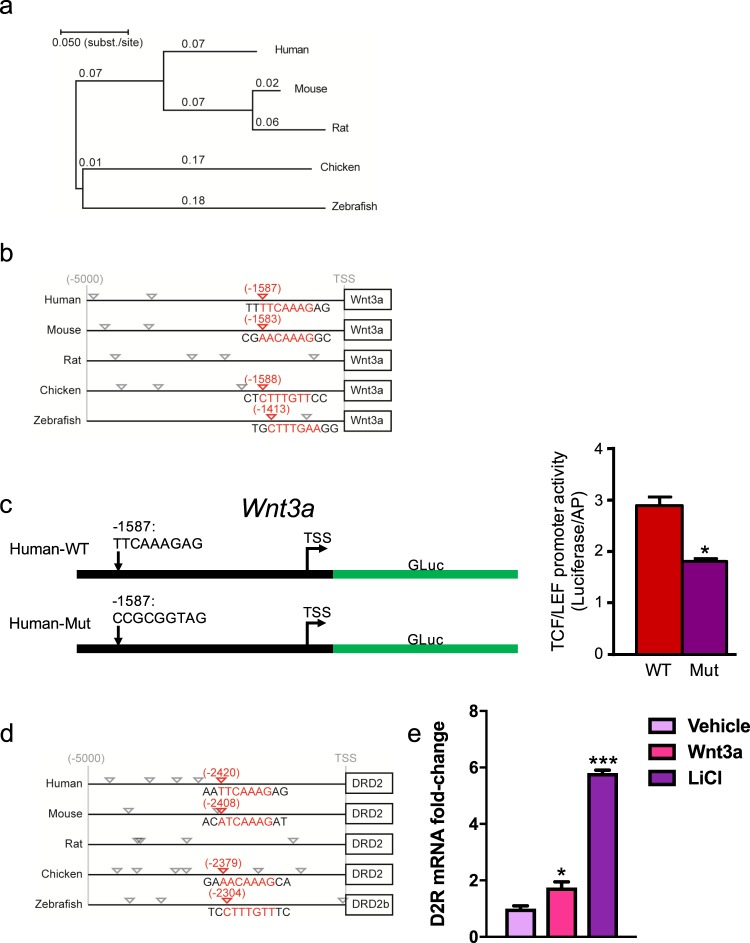
Modulation of Wnt3a expression via a conserved TCF/LEF promoter site. (**a**) Phylogenetic tree of *WNT3A* across multiple species: human, mouse, rat, chicken, and zebrafish. The *WNT3A* exons were used to construct the phylogenetic tree. Evolutionary distances in the units of base substitutions per site were calculated using the maximum composite likelihood method. The phylogenetic tree was then constructed using the Neighbor-Joining method with the sum of branch lengths = 0.645. **(b)** Binding site analysis of aligned human, mouse, rat, chicken, and zebrafish *WNT3A* promoter sequences highlighting conserved β-catenin-binding TCF/LEF sites within the 5 kb region upstream of the transcriptional start site (TSS). **(c)** Schematic representation of our luciferase reporter of TCF/LEF-dependent *WNT3A* transcription (left panel). The reporter construct consists of a selected segment of the human *WNT3A* promoter region containing a highly conserved TCF/LEF site upstream of the *Gaussia* luciferase gene. We show two variants of the reporter: one containing the wild-type (WT) TCF/LEF site (Human-WT) and another where the TCF/LEF site was inactivated by mutation (Human-Mut). Upon expression of a *WNT3A* transcriptional reporter in human renal proximal tubule cells, the Human-Mut reporter demonstrated significantly decreased *WNT3A* promoter activity compared with the WT control (right panel). The data were normalized to alkaline phosphatase expression. *P < 0.05; Student’s t-test. **(d)** Binding site analysis of aligned human, mouse, rat, chicken, and zebrafish *DRD2* promoter sequences highlighting conserved β-catenin-binding TCF/LEF sites within the 5 kb region upstream of the TSS. **(e)** Treatment with either LiCl (25 mM, 24 hr) or recombinant Wnt3a (100 ng/ml, 24 hr) significantly increased D2R mRNA expression. Data are expressed as mean ± SEM of 3 experiments conducted on separate experimental dates and performed in triplicate. *P < 0.05; Student’s t-test (**c**). *P < 0.05, ***P < 0.001; one-way ANOVA, Holm-Sidak post-hoc test (**e**).

In freshwater polyp *Hydra* species including *Hydra magnipapillata* and *Hydra vulgaris*, there is evidence suggesting that *Hydra Wnt3* (*HyWnt*) transcription is modulated by *cis*-regulation through β-catenin signaling^64^. The presence of a TCF/LEF autoregulatory element within the *HyWnt* promoter allows β-catenin to further increase HyWnt expression and thus potentiate Wnt signaling^64^. Based on D2R′s effects on β-catenin phosphorylation and TCF/LEF transcription, we asked whether similar *cis*-regulatory elements are used to regulate mammalian Wnt3a expression. To examine this possibility, we searched for potential TCF/LEF binding sites in the promoter region of the mammalian *WNT3A* gene. We constructed a phylogenetic tree of human *WNT3A* and discovered marked conservation across several species: human, mouse, rat, chicken, and zebrafish (Fig. 6a). Among the conserved regions, there were multiple TCF/LEF binding sites in the 5 kb promoter region upstream of the *WNT3A* transcriptional start site (TSS) of human, rat, mouse, and chicken *WNT3A* but only two TCF/LEF binding sites in zebrafish (Fig. 6b). Nevertheless, the zebrafish TCF/LEF site at position −1413 is well-conserved across all species examined except for the rat. These data suggest that, as in *Hydra*, Wnt3a expression may be regulated through β-catenin’s actions on conserved TCF/LEF binding sites within the *WNT3A* promoter. We therefore created a reporter of *WNT3A* transcription consisting of a portion of the *WNT3A* promoter that included the conserved TCF/LEF site fused to luciferase (Fig. 6c). We used the luminescence readout from the reporter to determine the potential contribution of this site to levels of *WNT3A* transcription by mutating the conserved TCF/LEF site to block β-catenin-mediated changes. We found that mutation of the TCF/LEF site significantly diminished *WNT3A* promoter activity (Fig. 6c). These results suggest that an important role for TCF/LEF sites within the *WNT3A* promoter may be to autoregulate Wnt3a expression. Such sites may therefore potentially contribute to D2R′s effects on Wnt3a expression, given D2R′s ability to modulate β-catenin activity.

Strikingly, our analyses of putative TCF/LEF sites revealed that the *DRD2* promoter also contains *cis*-regulatory TCF/LEF sites. We conducted a phylogenetic analysis of conserved TCF/LEF binding sites within the 5 kb promoter region upstream of the TSS of the human *DRD2* gene and its orthologs in mouse, rat, chicken, and zebrafish. Similar to *WNT3A*, along with several TCF/LEF sites within the respective promoter regions, there is a highly conserved TCF/LEF site evident in all species except for the rat (Fig. 6d). These results suggest that β-catenin may act as a convergence point to balance D2R and Wnt3a-driven signals.

We examined the functional relevance of the conserved TCF/LEF binding site within the *DRD2* promoter, predicting that stimulation of the Wnt3a signaling pathway would upregulate D2R expression. Indeed, we observed that treatment with either LiCl or recombinant Wnt3a protein, strong drivers of Wnt3a pathway signaling, significantly increased D2R mRNA expression (Fig. 6e).

### Increased D2R expression modifies cell proliferation in the ischemia/reperfusion-injury model

**Figure 7:**
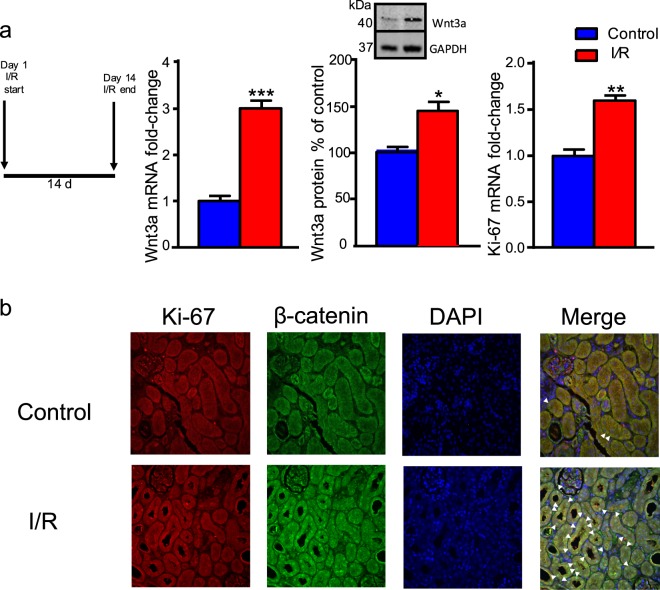
Wnt3a expression and cell proliferation increase in an ischemia/reperfusion-injury disease model. (**a**) Ischemia/reperfusion-injury (I/R) caused a 200% increase in Wnt3a mRNA levels (left panel) and 50% increase in Wnt3a protein levels (middle panel) in mouse renal cortex. There was an accompanying 55% increase in expression of the Ki-67 cell proliferation marker compared with the control (right panel). Alongside is the schedule for the I/R treatment. (**b**) Representative confocal immunofluorescence images of mouse renal cortex following I/R demonstrated increased nuclear Ki-67 (red), nuclear β-catenin (green), and increased nuclear co-localization of Ki-67 and β-catenin with DAPI (blue). Merged images: blue = DAPI, red = Ki-67, green = β-catenin, overlap = white); arrowheads indicate nuclear overlap among Ki-67, β-catenin, and DAPI signals. Scale bar = 50 µm. All data are represented as the mean ± SEM using 4 animals per group. All measurements were performed in triplicate. *P < 0.05, **P < 0.01, ***P < 0.001; Student’s t-test.

**Figure 8:**
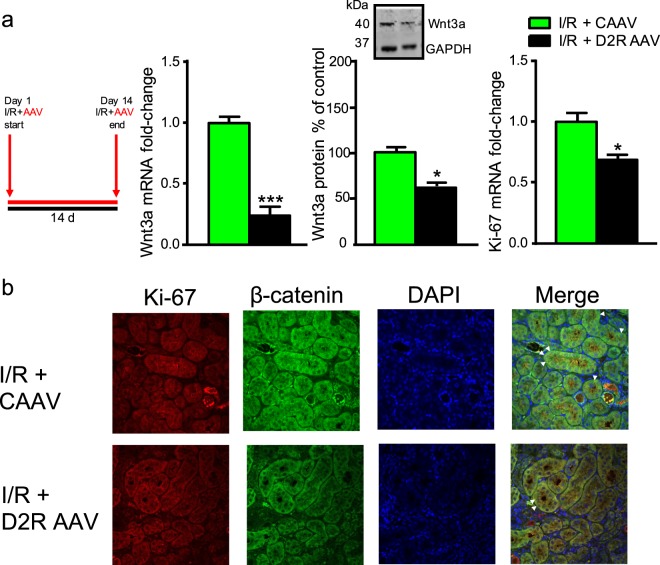
D2R overexpression partially reverses increased Wnt3a expression and cell proliferation in response to ischemia/reperfusion injury. (**a**) AAV-mediated D2R overexpression (D2R AAV) in mouse kidney prior to reperfusion caused a 70% decrease in Wnt3a mRNA levels (left panel) and a 40% decrease in Wnt3a protein levels (middle panel). There was also a 30% decrease in Ki-67 mRNA in the renal cortex relative to the control AAV vector (right panel). Alongside is the schedule for the respective ischemia/reperfusion (I/R) and AAV treatments. (**b**) Representative confocal immunofluorescence images of mouse renal cortex following AAV-mediated D2R overexpression in an I/R model of acute renal injury. There was decreased nuclear Ki-67 (red), nuclear β-catenin (green) and decreased nuclear co-localization of Ki-67 and β-catenin with DAPI (blue). Merged images: blue = DAPI, red = Ki-67, green = β-catenin, overlap = white); arrowheads indicate nuclear overlap among Ki-67, β-catenin, and DAPI signals. Scale bar = 50 µm. All data are represented as the mean ± SEM using 4 animals per group. All measurements were performed in triplicate. *P < 0.05, ***P < 0.001, Student’s t-test.

We investigated the relevance of D2R′s modulation of Wnt3a expression and cell proliferation within a disease context using the ischemia/reperfusion-injury (I/R) model of acute kidney injury. We chose this model system given the critical role played by cell proliferation in the associated pathology^65,66^. We observed a 3-fold increase in Wnt3a mRNA expression and 25% increase in Wnt3a protein expression following I/R, along with increased Ki-67 mRNA levels (Fig. 7a), consistent with established evidence of increased cell proliferation following reperfusion^66,67^. We also found increased nuclear co-localization between Ki-67 and β-catenin after reperfusion, demonstrating that the Wnt/β-catenin pathway is active in the proliferating cells (Fig. 7b). By contrast, AAV-mediated D2R overexpression immediately before the reperfusion period almost completely prevented the I/R-induced increase in Wnt3a mRNA and protein expression (Fig. 8a). Increasing D2R expression just prior to reperfusion also significantly diminished cell proliferation based on Ki-67 mRNA quantification (Fig. 8a). Confocal microscopy imaging of the D2R-overexpressing tissue showed a decrease in the nuclear co-localization between Ki-67 and β-catenin (Fig. 8b). These data further suggest an important link between D2R and Wnt signaling *in vivo*. Overall, our results show that boosting D2R signaling can negatively modulate Wnt3a-mediated cell proliferation induced by I/R and thereby modify one of the main contributors to pathologic change within this disease model.

## Discussion

Both DA and Wnt signaling pathways are nearly ubiquitous across organ systems and species. These signaling systems are integral drivers of physiological processes across development and have been implicated in a diverse array of diseases ranging from cancer to schizophrenia^15,29,57,63^. Yet, despite decades of study, many fundamental questions still remain concerning the mechanisms by which these two pathways are regulated and whether they intersect in specific cellular states or cell types. For example, though the sequence of signaling within the Wnt/β-catenin pathway has been thoroughly mapped out, the mechanisms regulating the expression of key Wnt ligands such as Wnt3a remain poorly understood. Similarly, downstream of the DA receptors, the precise physiological relevance of signaling via the G protein-dependent versus -independent pathways is largely unknown. Last but not least, despite sharing several common signaling components^15^, it has remained unclear whether DA and Wnt pathways modulate one another and, if so, under what contexts.

While DA has been studied primarily in the central nervous system, it also plays important functions in diverse peripheral organs including the kidney, pancreas, and gut^6,68,69^. These organs have the capacity to synthesize DA, which then signals locally in an autocrine and/or paracrine manner through DA receptors, including D2R^52,53,70^. In the pancreatic β-cell, D2R signaling is implicated as a negative modulator of insulin secretion^3,52^. In the kidney, renal DA regulates systemic blood pressure, in part, through D_2_-like receptor signaling^10,12^, via its effects on fluid and electrolyte balance as well as regulation of the secretion/metabolism of hormones such as aldosterone and angiotensin II^6,11,70^. Moreover, genetic hypertension is associated with alterations in DA production and D_2_-like receptor function^6^. Likewise, Wnt/β-catenin pathway signaling is essential in both kidney and pancreatic development and is implicated in the pathophysiology associated in both organs^71,72^.

Here, we present evidence that signaling through D2R modulates the Wnt pathway. We provide a putative mechanism for this crosstalk: D2R-dependent β-arrestin-2 signaling leads to diminished phosphorylation of β-catenin. This in turn promotes β-catenin’s translocation to the nucleus and increased TCF/LEF-mediated transcription within the Wnt/β-catenin pathway, including Wnt3a itself. In addition to G protein-dependent signaling, D2R also signals through β-arrestin-2, a protein long known to facilitate receptor internalization^23,73^. The β-arrestin-2 pathway is an important route for further downstream signaling involving AKT and GSK-3β kinases, critical signaling molecules implicated in numerous cellular functions including metabolism and proliferation^17,19–21,74^. Though the original β-arrestin-2 studies were conducted largely in rodent brain and *in vitro*, we show that these β-arrestin-2-dependent D2R signaling pathways are present in both human and rodent renal cells, suggesting that D2R′s capacity to modulate AKT phosphorylation and activity is a more general principle that is conserved across tissues and species. Our data also establish a model where D2R signaling via the β-arrestin-2-dependent pathway modulates TCF/LEF promoter activity through its ability to modify β-catenin’s degradation and capacity to translocate into the nucleus. This represents an important new mechanism by which GPCRs like D2R may utilize G protein-independent, β-arrestin-2-dependent signaling to modulate transcription via interactions with the Wnt/β-catenin pathway.

Analogous to the kidney, we found that D2R activity also modulates Wnt3a-dependent β-catenin transcriptional activity in INS-1E cells, a rat pancreatic β-cell line, suggesting that D2R′s ability to modulate Wnt signaling is conserved across both species and tissues. There is precedent for GPCR modulation of Wnt/β-catenin signaling in β-cells. Earlier work demonstrated that agonists of glucagon-like peptide-1 receptor, a GPCR, could enhance β-cell Wnt signaling^75^, ultimately leading to changes in β-cell mass through its actions on TCF/LEF-based transcription^75,76^. Additionally, Min and colleagues reported that the effects of D2R on neuronal Wnt signaling could also be agonist-independent and based, in part, on direct physical interactions between D2R and β-catenin^25^. This raises the possibility that at least some of this signaling is constitutive. Indeed, our data in renal cells using D2R blockade by sulpiride or siRNA-mediated D2R knockdown indicate some degree of constitutive *(i.e*., agonist-independent) regulation of Wnt signaling which is consistent with this earlier work^25^. Moreover, in addition to β-arrestin-2-dependent GPCR signaling, there is increasing evidence that inhibitory heterotrimeric Gα_i/o_ proteins are also involved in Wnt-induced responses^77–79^. Therefore, we propose that D2R stimulation may also trigger recruitment and activation of sufficient Gα_i/o_ to displace these proteins from FZD receptors, leading to a reduced G protein-dependent Wnt-stimulated response. Such a model may provide an additional explanation for our findings demonstrating that D2R stimulation by agonists including DA, quinpirole and bromocriptine reduces Wnt-dependent signaling in β-cells.

We further dissected D2R-mediated effects on TCF/LEF transcriptional activity in human renal proximal tubule cells, taking advantage of *DRD2* SNPs that negatively regulate endogenous D2R expression and function^13,80–82^. Several SNPs within the human *DRD2* gene have been associated with clinically relevant conditions including elevated blood pressure and essential hypertension^13^. Moreover, a recent study in an Asian Indian population with type 2 diabetes found that a SNP that decreased D2R expression also conferred susceptibility to chronic diabetic nephropathy^83^. Similarly, the combination of two highly prevalent *DRD2* SNPs, rs6276 and rs6277, decreased D2R expression as a consequence of diminished D2R mRNA stability as well as via decreased receptor availability and affinity^13,80–82^. We found that the combined effects of these two *DRD2* SNPs also diminished levels of phosphorylated β-catenin, presumably freeing more β-catenin to successfully evade degradation and thus to stimulate transcription of Wnt/β-catenin pathway target genes. Given the Wnt/β-catenin pathway’s roles in mediating cell proliferation, our results suggest that D2R signaling may utilize such an intersection with Wnt signaling to modulate β-catenin and Wnt-dependent cell proliferation. Consistent with this, recent studies suggest that D_2_-like receptor activation inhibits cellular proliferation while, conversely, its inhibition increases cellular proliferation^84–87^. Furthermore, in pancreatic β-cells, pharmacological D2R inhibition not only increases cell proliferation, but also decreases cell de-differentiation, collectively leading to increased β-cell mass^85^. Given D2R′s ability to modify β-catenin-mediated TCF/LEF transcription, we propose that the interconnections between D2R and Wnt signaling may be critical in modulating cell proliferation under physiological circumstances, at least in the cell types we assayed (human and mouse renal proximal tubule cells and rat pancreatic β-cells [INS-1E]). Moreover, D2R′s inhibitory effects on proliferation may be a regulatory mechanism by which cells avoid uncontrolled expansion.

Given the interconnections between D2R and Wnt/β-catenin signaling pathways, we investigated whether Wnt3a, one of the primary ligands for the Wnt receptor FZD (FZD_2_ in kidney), is also implicated in these processes. Indeed, Wnt3a has been shown to mediate cell proliferation^88,89^. We used several complementary approaches to demonstrate significant increases in Wnt3a expression in response to reduced D2R expression including D2R knockdown in mouse renal proximal tubule cells *in vitro* and renal cortex *in vivo*, as well as human renal proximal tubule cells where D2R expression is reduced by the presence of SNPs. Together, our results suggest an important relationship between D2R signaling and Wnt3a expression. Furthermore, these data describe a possible new mechanism where alterations in D2R expression may trigger a corresponding change in Wnt3a levels which then alters β-catenin-mediated TCF/LEF transcription in processes including cell proliferation.

Prior work in Hydra demonstrated that TCF/LEF sites within the promoter of the Hydra Wnt3a ortholog, *HyWnt3*, regulated Wnt3 expression^64^. Nevertheless, it remained unclear whether such a regulatory mechanism for Wnt3a expression was generalized across species, especially in mammals. We now show that the promoters corresponding to Wnt3a orthologs contain numerous TCF/LEF sites across multiple species from zebrafish to mice and humans. Our findings demonstrate remarkable conservation of these regulatory elements across species and suggest β-catenin is a positive modulator of Wnt3a expression. However, this also leads to the question: how do cells terminate such a positive feedback mechanism for Wnt3a expression to avoid uncontrolled Wnt3a signaling that may ultimately be detrimental to overall cellular viability and/or lead to uncontrolled cell proliferation?

**Figure 9:**
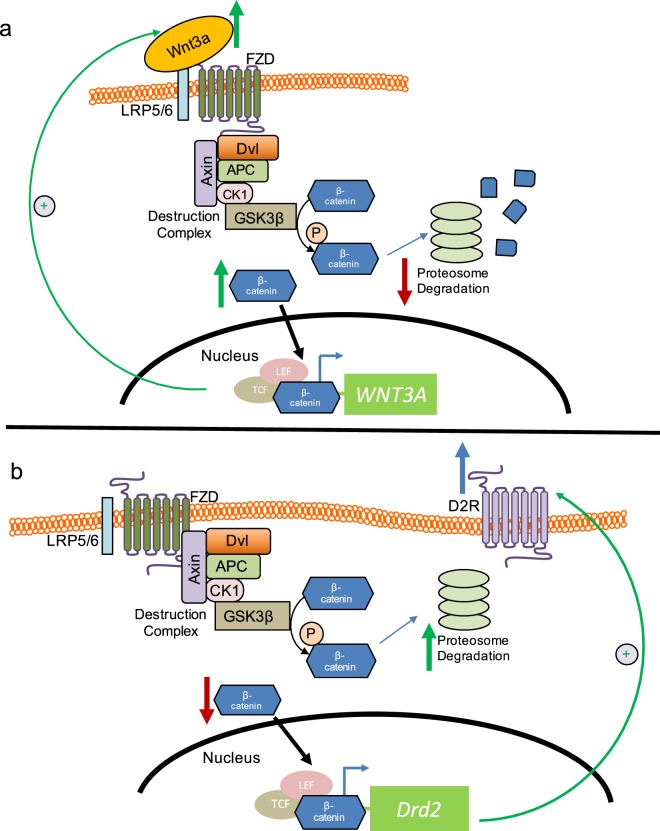
Model of cross-talk between D2R and Wnt/β-catenin pathways, and Wnt3a transcriptional regulation. We propose the following mechanism by which D2R and Wnt3a reciprocally regulate their expression: **(a)** Wnt3a stimulation of the Frizzled (FZD) receptor/LRP5/6 complex leads to less β-catenin degradation via the destruction complex and proteasomal pathway. Instead, more β-catenin is available for translocation into the nucleus where it increases Wnt3a transcription and expression via a TCF/LEF site within the Wnt3a promoter. This leads to a positive feedback loop that amplifies further Wnt3a signaling. **(b)** Increased nuclear translocation of β-catenin also upregulates D2R expression through TCF/LEF sites within the D2R promoter. Increased D2R expression leads to more β-catenin phosphorylation, resulting in enhanced β-catenin proteasomal degradation and therefore less β-catenin nuclear translocation. This ultimately inhibits further β-catenin-mediated Wnt3a transcription to maintain cellular homeostasis.

We propose that D2R may contribute to the regulatory mechanisms that limit Wnt3a′s capacity to induce its own increased expression and thus prevent uncontrolled cell proliferation. One potential mechanism may be D2R′s ability to negatively regulate β-catenin nuclear translocation via its actions on AKT and GSK3β. With our discovery that D2R itself contains evolutionarily-conserved TCF/LEF sites in its promoter, this suggests a model where D2R expression may also be promoted following an initial Wnt3a-induced boost in β-catenin-mediated transcription (Fig. 9). We confirmed this model, showing that direct stimulation of the Wnt3a pathway via treatment with recombinant Wnt3a or LiCl causes significant increases in D2R transcription. We assert that this rise in D2R levels dampens subsequent increases in Wnt3a levels and thus keeps processes such as cell proliferation in check. Such a model of reciprocal D2R and Wnt3a signaling at the transcriptional level establishes the importance of D2R signaling as a novel modulator of Wnt/β-catenin signaling under physiological conditions (Fig. 9). Furthermore, our data showing that LiCl treatment significantly increases D2R expression may be relevant not only in renal cells but also in neurons, suggesting a new mechanism for LiCl’s action in the central nervous system.

We also investigated a potential role for renal D2R signaling in a disease context via the I/R model of acute kidney injury^65,66,90^. Classically, induction of renal ischemia followed by reperfusion leads to increased cell proliferation and subsequent development of injury, hypertrophy and decreased overall renal function^4,65,67^. Significantly, we found that acute renal injury in the I/R model increased Wnt3a expression and led to increased nuclear localization of both Ki-67 and β-catenin in injured tissue, indicating the involvement of the Wnt/β-catenin pathway in injury-induced cell proliferation. Moreover, AAV-mediated overexpression of D2R diminished this injury-induced cell proliferation and reduced the increase in Wnt3a expression within the injured tissue. Our data are consistent with earlier work implicating elevated Wnt3a expression in renal injury^91^. This is also consistent with our previous results showing that increasing renal D2R decreases the extent of ischemic injury, ameliorating both renal function and normalizing blood pressure^4^. Conversely, silencing of D2R was itself sufficient to induce renal injury as demonstrated by increased levels of kidney injury marker KIM-1, and this effect was reversed by D2R overexpression^4^. These data further suggest the importance of renal D2R signaling in maintaining normal renal physiology, as well as in acute renal injury. Together, our results suggest that D2R signaling and its interactions with the Wnt/β-catenin pathway may play critical roles in modulating the extent of renal injury and that modifying D2R expression may serve as a novel therapeutic avenue for preventing the progression of kidney disease.

At present, much of our understanding of D2R and GPCR signaling more generally derives from its acute effects on downstream G proteins. While G protein-dependent signaling is often rapid, occurring over seconds and minutes, here we show that the G protein-independent, β-arrestin-2-dependent arm of D2R signaling may also play important roles in cellular physiology and pathophysiology on a longer time scale via its effects at the transcriptional level. This suggests a paradigm where D2R utilizes multiple highly conserved downstream signaling pathways to selectively induce both acute changes in signaling and concurrent longer-term changes via its effects on transcriptional regulation. Such GPCR-modulated effects on transcription may be quite relevant physiologically to regulate cell proliferation or differentiation during development as well as in the context of disease states including diabetes or hypertension. There is precedent for GPCR regulation of transcription through the receptors’ effects on intracellular cAMP levels via G protein-dependent signaling^92^. By modulating cAMP production through recruitment of G proteins such as Gα_s_ or Gα_i_, GPCRs may change the activity of cAMP-dependent gene transcription factors including cAMP response element binding proteins (CREB-BPs), and thus alter levels of gene transcription^93^. Such a mechanism may have direct implications for β-catenin-mediated transcription since CREB-BPs are also constituents of the β-catenin/TCF transcriptional complex^94^. Therefore, for a Gα_i/o_-coupled receptor like D2R, G protein and cAMP-dependent signaling may synergize with D2R′s effects on β-catenin phosphorylation to ultimately determine the efficacy of β-catenin/TCF/LEF-mediated transcriptional regulation.

## Conclusions

We show that D2R modulates signaling through the Wnt/β-catenin pathway, in part, through its effects on Wnt3a expression. Our work presents a novel mechanism by which a GPCR, D2R, works through the β-arrestin-2-dependent pathway to modulate transcription. Furthermore, given the ability of D2R-blocking drugs, such as antipsychotic medications to induce metabolic syndrome consisting of disturbances such as diabetes and hypertension^95^, our findings may shed important new light on the underlying mechanisms responsible for these profound and previously unanticipated effects on cellular physiology. These studies therefore open the door to a broader understanding of intracellular signaling and its implications in health and disease.

## Materials and Methods

### Compounds

The drugs used in the present study were purchased from Sigma-Aldrich (St. Louis, MO), unless indicated otherwise: plasmocin (Fisher Scientific, Hampton, NH); EGF, dexamethasone, triiodothyronine, insulin-transferrin-selenium (ITS, 1X, Invitrogen, Carlsbad, CA); penicillin/streptomycin (1X, Invitrogen); fetal bovine serum (Invitrogen); amphotericin (Gibco Thermo Fisher Scientific, Gaithersburg, MD), and G418 (Millipore-Sigma-EMD, Burlington, MA), (−)-quinpirole hydrochloride, (S)-(−)sulpiride, LiCl, bromocriptine mesylate, L-glutamine, HEPES, sodium pyruvate, and 2-mercaptoethanol.

### Cell culture

Undifferentiated mouse cells were cultured from progenitor kidney cells isolated from mouse embryo kidneys following the procedure described by Woost *et al*.^96^; cells were kindly supplied by Dr. Ulrich Hopfer (Case Western Reserve School of Medicine, Cleveland, OH). These cells were differentiated into mature mouse renal proximal tubule cells (mRPTCs) and cultured to 60–70% confluence. mRPTCs were maintained in DMEM-F12 (Invitrogen) supplemented with fetal bovine serum (10%), penicillin/streptomycin (1X) and amphotericin (2.5 μg/ml). The mRPTCs were subsequently transfected with either vehicle (transfection reagent, Hyperfect, Qiagen, Valencia, CA), non-silencing AllStars Negative Control siRNA (20 nM, catalog#1027281, Qiagen) or mouse D2R siRNA (20 nM, catalog#SI04918410, Qiagen) and studied 72 hr post-transfection. For other experiments, mRPTCs were cultured to 90–95% confluence, serum-starved for 2 hr and treated for an additional 24 hr in serum-free medium with vehicle (PBS) or drug (1 μM quinpirole or 1 μM sulpiride).

Human renal proximal tubule cells (hRPTCs) were isolated from human kidney specimens obtained solely from male patients who had unilateral nephrectomy due to renal carcinoma or trauma. All samples were completely de-identified. Informed consent was obtained from all subjects for collection of these specimens. All procedures strictly followed the guidelines of a University of Virginia institutional review board-approved protocol, as well as the Declaration of Helsinki.

For isolation of the hRPTCs, only the healthy renal pole (as judged by visual and histological inspection) distal from the affected part of the kidney was used; cells were subsequently immortalized, as previously described^97^. Immortalized hRPTCs were maintained at 37°C, 5% CO_2_ and cultured to 90–95% confluence in non-polarizing conditions in DMEM-F12 (Invitrogen) containing (final concentrations): plasmocin (2.5 μg/ml), EGF (10 ng/ml), dexamethasone (36 ng/ml), triiodothyronine (2 ng/ml), insulin-transferrin-selenium (ITS, 1X), penicillin/streptomycin (1X), fetal bovine serum (2%), and G418 (0.4%). The hRPTCs were also genotyped for the presence of rs6276, rs6277 *DRD2* SNPs. Four cell lines from subjects bearing rs6276 and rs6277 *DRD2* SNP alleles as well as four control subjects without these SNPs (D2R-WT) were studied. These two SNPs occur within and adjacent to the *DRD2* gene. Because of their close proximity, they are likely to be in linkage disequilibrium which limits the possibility of identifying cell lines bearing only one of the SNPs. hRPTCs were transfected with either vehicle (transfection reagent, Hyperfect, Qiagen, Valencia, CA), non-silencing AllStars Negative Control siRNA (20 nM; catalog#1027281, Qiagen), human D2R siRNA (20 nM; catalog#SI03076479, Qiagen), or human Wnt3a siRNA (20 nM; catalog#SI00145565, Qiagen) and studied 72 hr post-transfection.

To test the effects of Wnt/β-catenin pathway activation on D2R mRNA expression, hRPTCs were incubated in serum-free medium in the presence or absence of either recombinant Wnt3a protein (100 ng/ml, 24 hr) (catalog#G3154; LifeSpan Biosciences, Inc., Seattle, WA), LiCl (25 mM, 24 hr) or vehicle controls. mRNA was extracted from the respectively treated cells and cDNA was subsequently prepared as described below. Changes in D2R expression were measured by quantitative real-time PCR.

INS-1E cells (gift of Pierre Maechler, University of Geneva) were maintained in a humidified 37 °C incubator with 5% CO_2_. The cells were cultured as described earlier^51^ with RPMI 1640 media (Life Technologies, Norwalk, CT), supplemented with 5% (v/v) heat inactivated fetal bovine serum, 2 mM L-glutamine, 10 mM HEPES, 1 mM sodium pyruvate, 100 units/mL penicillin, 100 μg/mL streptomycin, and 50 μM 2-mercaptoethanol. The cells were subcultured weekly in a ratio of 1:5 to 1:10 with medium renewal every 3 to 4 days.

### Preparation of Wnt3a-conditioned and L-cell control medium

Mouse fibroblast-derived L-Wnt3A cells (CRL-2647; ATCC, Manassas, VA) that stably express and secrete Wnt3a and the control untransfected parental L-Cell line (CRL-2648, ATCC) were purchased from ATCC (Manassas, VA). Control L-cells were cultured in Dulbecco’s Modified Eagle’s Medium (Thermo Fisher Scientific) supplemented with 10% (v/v) fetal bovine serum. L-Wnt3A cells were supplemented with the same media as control L-cells along with the addition of 0.4 mg/ml G-418. Both cell lines were subcultured in a ratio of 1:10 with medium renewal every 2 to 3 days.

### Transient transfection

hRPTCs were transfected with an empty vector or plasmid harboring wildtype human *DRD2* under the control of cytomegalovirus promoter (RC202476; Origene Technologies, Inc., Rockville, MD), using the Fugene HD transfection reagent (E2311; Promega Corporation, Madison, WI) as per the manufacturer’s guidelines. Briefly, 4 μg of plasmid DNA were diluted to 400 μl with serum-free medium and mixed with 16 μl of Fugene HD transfection. The transfection complex was incubated for 15 min at room temperature and added dropwise onto the cells in 10 cm tissue culture plates. Three days following transfection, the hRPTCs were harvested for protein extraction and immunoblotting, total RNA preparation, and RT-qPCR, or processed for immunofluorescence staining. Transfection efficiency was 40–45%. Transfections in INS-1E cells were performed using Lipofectamine 2000 transfection reagent (Invitrogen) overnight according to manufacturer instructions.

### AAV vectors

To construct D2R AAV, a 1.7-kb human *DRD2* cDNA from plasmid RC202476 (Origene Technologies Inc.) was excised as a KpnI and FseI fragment and inserted in between the same restriction sites within the pACS plasmid^98^. The control AAV (CAAV) vector, which does not encode any protein, contains an EGFP cDNA in reverse orientation under the control of the CMV promoter. Recombinant AAV vector genomes were subsequently packaged into capsids from the AAV-9 serotype, via the triple transfection method in HEK 293 cells, as described previously^99,100^.

### TCF/LEF reporter assay

mRPTCs were treated with *Drd2*-specific siRNA (D2R siRNA; 20 nM, Qiagen) or non-silencing siRNA (NS siRNA; 20 nM, Qiagen). After 48 hr, the mRPTCs were trypsinized and seeded for transient reverse transfection with a TCF/LEF luciferase reporter provided by the Cignal Reporter Assay TCF/LEF luciferase reporter system (SABiosciences-Qiagen). Following transfection, the mRPTCs underwent drug treatment with either quinpirole (1 μM) or vehicle for 24 hr; acute sulpiride treatment (1 μM) was for 6 hr. Acute recombinant Wnt3a treatment (LifeSpan Biosciences, Inc., Seattle, WA; 100 ng/ml) was for 2 hr. INS-1E cells were similarly reverse-transfected using the Cignal Reporter Assay TCF/LEF luciferase reporter system, followed by treatment with either RPMI 1640 or Wnt3a-conditioned medium, in the presence or absence of the D2R agonist bromocriptine (10 µM) for 18 hr.

### RNA extraction and cDNA preparation

Kidney samples or cell lysates were homogenized, and total RNA was extracted with Trizol (Invitrogen, Carlsbad, CA) and further purified using the RNeasy RNA Extraction Mini kit (Qiagen). RNA samples were converted into first strand cDNA using an RT^2^ First Strand kit, following the manufacturer’s protocol (SABiosciences-Qiagen).

### Quantitative Real-Time PCR

Total RNA was purified using the RNeasy Plus Mini kit (Qiagen, Valencia, CA). cDNA was prepared using an RT2 First Strand kit as per the manufacturer’s protocol (SABiosciences-Qiagen). Quantitative gene expression was analyzed by real-time PCR, performed on an ABI Prism 7900 HT (Thermo Fisher, Pittsburgh, PA). The assay used SYBR Green Real-Time PCR detection method (Qiagen) and all gene-specific primer assays were commercially available in the RT^2^ qPCR Primer Assay format, (SABiosciences-Qiagen) following manufacturer’s instructions. Primers used were: MKi-67 (catalog#PPM03457B); Wnt3a (catalog#PPM04720C); D2R (catalog#PPM04288A); β-arrestin-2 (catalog#PPM04814G); Gapdh (catalog#PPM02946E); all primers were commercially available from Qiagen. Data were analyzed using the ΔΔCt method and expressed as fold-change relative to control^101^. In the case of quantitation of β-arrestin-2 expression in control mouse and human renal samples, we specifically measured mRNA levels in arbitrary units in order to directly compare β-arrestin-2 levels of expression relative to the Gapdh control (Supplementary Fig. S1).

### Immunoblotting

Flash-frozen tissue homogenates and cell lysates were subjected to immunoblotting. The samples were homogenized or lysed in RIPA buffer, and equal amounts of protein (20 μg) were electrophoresed under reducing conditions on 4–20% gradient polyacrylamide gels and transferred onto nitrocellulose membranes. The primary antibodies used were: anti-phospho-AKT (T308 phosphorylation site; catalog#13038, Cell Signaling Technology, Danvers, MA); anti-AKT (catalog#4685, Cell Signaling Technology); anti-phospho-GSK3β (S9 phosphorylation site; catalog#5558, Cell Signaling Technology); anti-GSK3β (catalog#9832, Cell Signaling Technology); anti-phospho-β-catenin (S675 phosphorylation site; catalog#4176, Cell Signaling Technology); anti-β-catenin (catalog#8480, Cell Signaling Technology); anti-D2R (catalog#AB5084P, Millipore Sigma); anti-Wnt3a (catalog#2391, Cell Signaling Technology); anti-Gapdh (catalog#AB2302, Sigma); anti-tubulin (catalog#ab6046, Abcam, Cambridge, MA). Results were normalized to Gapdh or tubulin and expressed as percentage- or fold-change, relative to the average signal intensity obtained from the respective controls.

### Immunohistochemistry

The procedures for immunohistochemistry were described earlier^4^. Briefly, mouse kidneys were collected and fixed in 3.7% paraformaldehyde for 1-hr at 4°C. After washing in phosphate-buffered saline (3 times, 5 min each), the tissues were equilibrated with 30% sucrose in phosphate-buffered saline overnight. Paraffin-embedded tissue sections were immunostained for expression of Wnt3a using a rabbit monoclonal anti-Wnt3a antibody (catalog#ab172612, Abcam). We stained for Ki-67, a marker of cell proliferation, using a rabbit polyclonal anti-Ki-67 antibody (catalog#ab15580, Abcam). We also stained for β-catenin using a goat polyclonal anti-β-catenin antibody (catalog#AF1329, R&D Systems, Minneapolis, MN). DAPI was used to visualize the nuclei of individual cells. Secondary antibodies included donkey anti-rabbit Alexa Fluor 555 (catalog#A-31572; Invitrogen) and a donkey anti-goat Alexa Fluor 488 (catalog#A-11055; Invitrogen). The cells were counted in at least 3 microscopy fields from each section at 10x magnification (n ≥ 3 per group). Confocal images of immunostained tissue were acquired at 20x magnification using a spinning disk confocal microscope (Zeiss, Thornwood, NY).

### Cell proliferation assay

Cell proliferation was assessed using the Delfia Cell Proliferation kit (Caliper PerkinElmer Life Sciences, Waltham, MA). Approximately 1 × 10^4^ cells were seeded into a 96-well Isoplate (PerkinElmer Life Sciences) and grown in complete media. Bromodeoxyuridine (BrdUrd, 10 μM) was added to the medium 24 hr prior to the end of the assay. The cells were subsequently fixed, the DNA was denatured, and the amount of incorporated BrdUrd was determined using an Eu-labeled monoclonal anti-BrdU antibody. Following formation of highly fluorescent chelates, fluorescent signal was detected by time-resolved fluorometry using a Victor^3^ multilabel reader (PerkinElmer Life Sciences). The same number of cells was seeded on a separate 96-well Isoplate and grown overnight before fixation with 4% paraformaldehyde and nuclear staining with DAPI. DAPI fluorescence was detected also using a Victor^3^ multilabel reader. Final results are reported as Eu fluorescence normalized to DAPI signal.

### Animal procedures

All animals used in the study were C57BL/6 male mice (8–10 weeks old, weighing ~20 g) which were purchased from The Jackson Laboratory (Bar Harbor, ME). All animals were housed and handled in accordance with all appropriate NIH guidelines. Our experimental protocol was approved by the institutional review board and ethics committee of George Washington University (IACUC protocol#A353). For all experiments, we abided by all relevant and appropriate animal care guidelines and regulations including the ARRIVE guidelines. All efforts were made to ameliorate animal suffering.

### *In vivo* siRNA treatments

Mice were anesthetized with an intraperitoneal injection of pentobarbital sodium (50 mg/kg) and placed in a supine position. The mice were treated with NS siRNA or D2R siRNA by subcapsular infusion (3 μg/day) using osmotic minipump (ALZET Osmotic Pumps, Cupertino, CA) for 28 days in the left kidney, leaving the contralateral kidney intact^102^. The siRNA was loaded into the osmotic minipump with a polyethylene tube connected to the osmotic minipump. The tube was inserted underneath the kidney capsule for continuous siRNA delivery. A detailed description of the protocols used was provided previously^4^.

### Ureteral AAV infusions

For retrograde ureteral infusion of AAV vectors, 14 days following the initiation of siRNA treatment, the mice were anesthetized and operated on to expose the kidneys^4^. The distal ureteral portions closest to the bladder and the renal artery supplying the target kidney were clamped off using a microvenous clip. The ureter was punctured using a tuberculin syringe fitted with a 33-gauge needle and urine was gently aspirated out. A tuberculin syringe was placed into the ureter containing ~100 μl of the respective AAV vector (1 × 10^11^ viral genome particles) and the solution was retrogradely infused into the ureter. The needle was withdrawn and a microvenous clip was placed proximal to the injection site on the ureter to prevent leakage and to attain maximum exposure to the infusion. Fifteen minutes later, the arterial and ureteral clips were sequentially removed, and the ureter was inspected for any evidence of leakage. The abdominal contents were replaced in reverse order and the incision site was closed.

### Renal ischemia/reperfusion-injury model

The mice were anesthetized as above. Following exposure of the kidneys, both right and left renal vessels (artery and vein) were clamped with arterial clamps for 45 min to stop blood flow to both kidneys^4^. In control animals, while the kidneys were exposed, the right and left renal vessels were not clamped. Upon clamp removal, a subset of the previously clamped mice were subjected to bilateral retrograde ureteral infusion of D2R AAV (experimental condition), as described above; the control group in this case consisted of clamped mice injected with control AAV (CAAV). The abdominal incision was closed, and the mice were allowed to recover. Fourteen days later, the mice were euthanized with an overdose of pentobarbital sodium (100 mg/kg). The kidneys, and other selected organs were harvested.

### β-catenin and TCF/LEF binding site analysis

On the basis of the following sequence recognized by β-catenin and several TCF/LEF transcription factors (LEF1, TCF7, TCF7L1, TCF7L2): 5′-(A/T)(A/T)CAAAG-3′ (Wnt response element, WRE), we looked for conserved β-catenin-TCF/LEF binding sites upstream of *WNT3A* gene orthologs in 5 species that have an annotated *WNT3A* gene in the UCSC genome browser: Human (hg19), Mouse (mm10), Rat (rn6), Chicken (galGal5), and Zebrafish (danRer11). The 5-kb promoter region upstream of *WNT3A* TSS for each of the five species was searched for the eight possible WRE binding site sequences, 4 on each strand, in all available promoter isoform variants using a custom-made Python script. The same analysis was then extended to promoter regions 5-kb upstream of the *DRD2* TSS, comparing the same five species for conserved binding sites: Human, Mouse, Rat, Chicken, and Zebrafish. Specifically, for the *DRD2* ortholog in the danRer11 Zebrafish assembly, two different genes are listed in the UCSC genome browser: *DRD2a* and *DRD2b*. We found a conserved binding site corresponding to *DRD2b* which is reported in this study.

### *WNT3A* promoter activity assay

*WNT3A* promoter activity was measured using the Secrete-Pair Dual Luminescence Assay System (GeneCopoeia, Rockville, MD). The plasmid pWT/Wnt3a/GLuc contains a 1,721-base region from the human *WNT3A* promoter [−1635 to +86 of transcription start site (TSS)] driving the expression of naturally-secreted *Gaussia* luciferease (GLuc), and a secondary reporter with a CMV promoter driving the expression of secreted Alkaline Phosphatase (SEAP), serving as an internal control. The plasmid pMut/wnt3a/GLuc carries a mutation in which the conserved TCF/LEF binding site 5′-TTCAAAG-3′ at the position −1587 bases upstream of the TSS was mutated to 5′-CCGCGGT-3′. hRPTCs were co-transfected with NS siRNA (20 nM) or D2R siRNA (20 nM), and pWT/wnt3a/GLuc or pMut/wnt3a/GLuc. Two days later, hRPTCs were treated with fresh medium for 16 hr after which the cell culture supernatants were collected, and the luciferase activity measured via the Secrete-Pair Dual Luminescence Assay Kit (GeneCopoeia, Rockville, MD). Luciferase activity results were expressed as relative light units (RLUs) normalized to SEAP activity (RLUs/SEAP).

### Phylogenetic tree analysis

We used the Wnt3a protein coding regions (exon sequences) of human, mouse, rat, chicken, and zebrafish species to construct a phylogenetic tree using the MEGA-7 software package. The evolutionary distances in the units of base substitutions per site were calculated using the maximum composite likelihood method. There was a total of 1,052 positions after eliminating positions containing gaps and missing data. The phylogenetic tree was then constructed using the Neighbor-Joining method with the sum of branch lengths = 0.645.

### Statistics

All results are expressed as mean ± SEM. Comparisons between experimental groups were performed using Student’s t-tests for two groups or ANOVA followed by post-hoc Holm-Sidak analyses for more than two groups. All statistical analyses were performed using the GraphPad Prism software package (GraphPad Software, La Jolla, CA).

## Supplementary information

Supplementary Information

Dataset 1

## Data Availability

The data and reagents generated and/or analyzed during the current study are available from the corresponding authors upon reasonable request.
