# Supplementary material for: Dopamine D2 receptor modulates Wnt expression and control of cell proliferation
Source: Sci Rep. 2019 Nov 14;9:16861. doi: 10.1038/s41598-019-52528-4 (PMC6856370; doi:10.1038/s41598-019-52528-4)
Supplement: Supplementary file 1 — Supplementary Information [file 41598_2019_52528_MOESM1_ESM.pdf]

Supplementary Information

Dopamine D_2 receptor modulates Wnt expression and control of cell proliferation

Fei Han[1%], Prasad Konkalmatt[1%], Chaitanya Mokashi[2], Megha Kumar[1], Yanrong Zhang[1], Allen Ko[3], Zachary J. Farino[4], Laureano D. Asico[1], Gaosi Xu[1], John Gildea[5], Xiaoxu Zheng[1], Robin A. Felder[5], Robin E.C. Lee[2], Pedro A. Jose[1,6], Zachary Freyberg[4,7,§*], Ines Armando[1§*]

From the [1]Department of Medicine, School of Medicine and Health Sciences, The George Washington University, Washington DC, 20052; [2]Department of Computational & Systems Biology, University of Pittsburgh, Pittsburgh, PA 15213; [3]Institute of Human Nutrition, College of Physicians & Surgeons, Columbia University, New York, NY 10032; [4]Department of Psychiatry, University of Pittsburgh, Pittsburgh, PA 15213; [5]Department of Pathology, The University of Virginia, Charlottesville, VA 22904; [6]Department of Pharmacology and Physiology, School of Medicine and Health Sciences, The George Washington University, Washington DC, 20052; [7]Department of Cell Biology, University of Pittsburgh, Pittsburgh, PA 15213

[%]Authors contributed equally

[§]Co-corresponding authors

* Corresponding Authors:

Zachary Freyberg, MD, PhD
University of Pittsburgh
3811 O'Hara Street
BST, W1640
Pittsburgh, PA 15213
Tel: 646-595-8317
freyberg@pitt.edu

Ines Armando, PhD
The George Washington University
2300 Eye Street
Ross Hall Suite 738
Washington, D.C. 20037
Tel: 202-994-0159
iarmando@gwu.edu

List of included materials: **Supplementary Figures:**

Figures S1, S2, S3, S4, S5, S6, S7, S8, S9

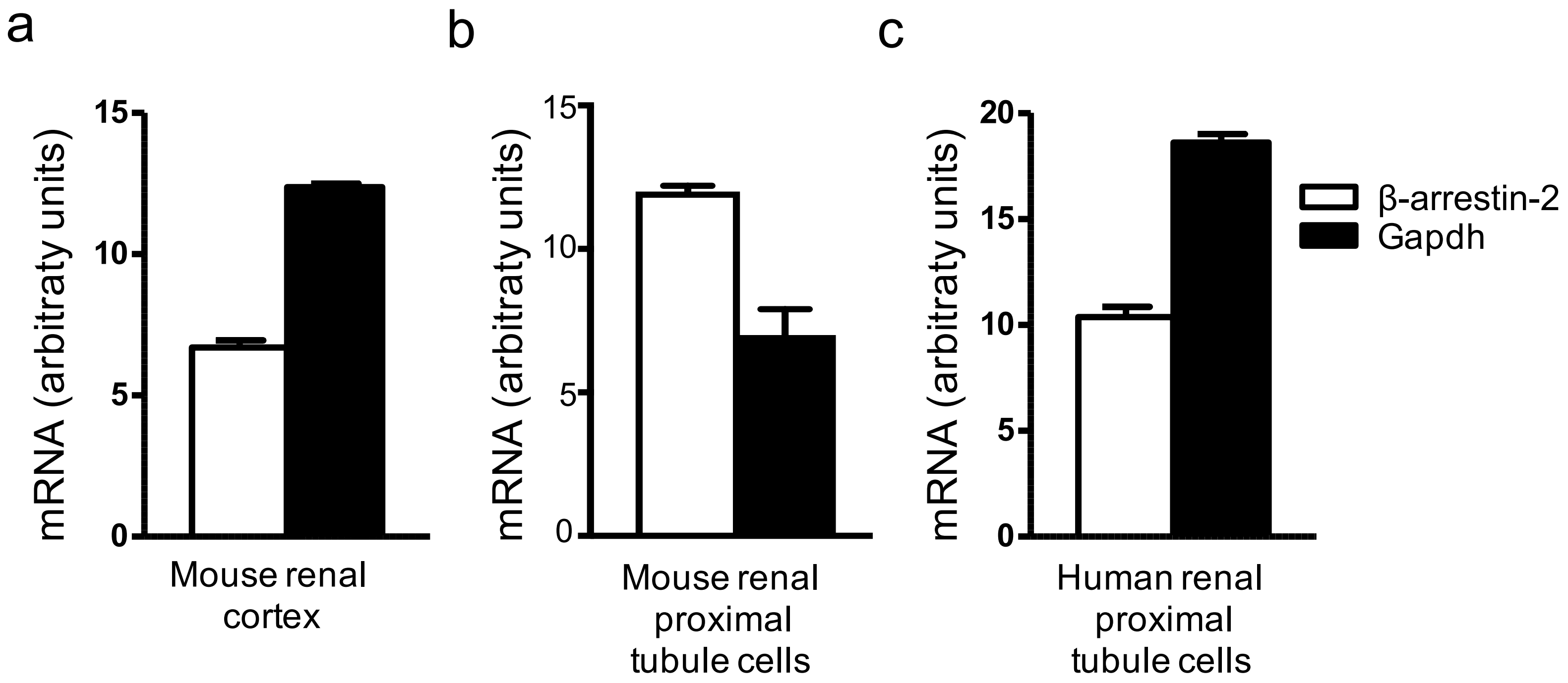

Supplementary Figure S1. Comparison of β-arrestin-2 mRNA expression relative to Gapdh in mouse and human renal proximal tubule cells.

The expression of β-arrestin-2 mRNA was determined by quantitative RT-PCR in samples obtained from mouse renal cortex (Panel a; n=4), mouse renal proximal tubule cells (Panel b; n=5-6) and human renal proximal tubule cells (Panel c; n=5). β-arrestin-2 expression was compared with that of the internal control, Gapdh, expressed in both mouse and human renal proximal tubule cells. Data are represented as the mean ± SEM.

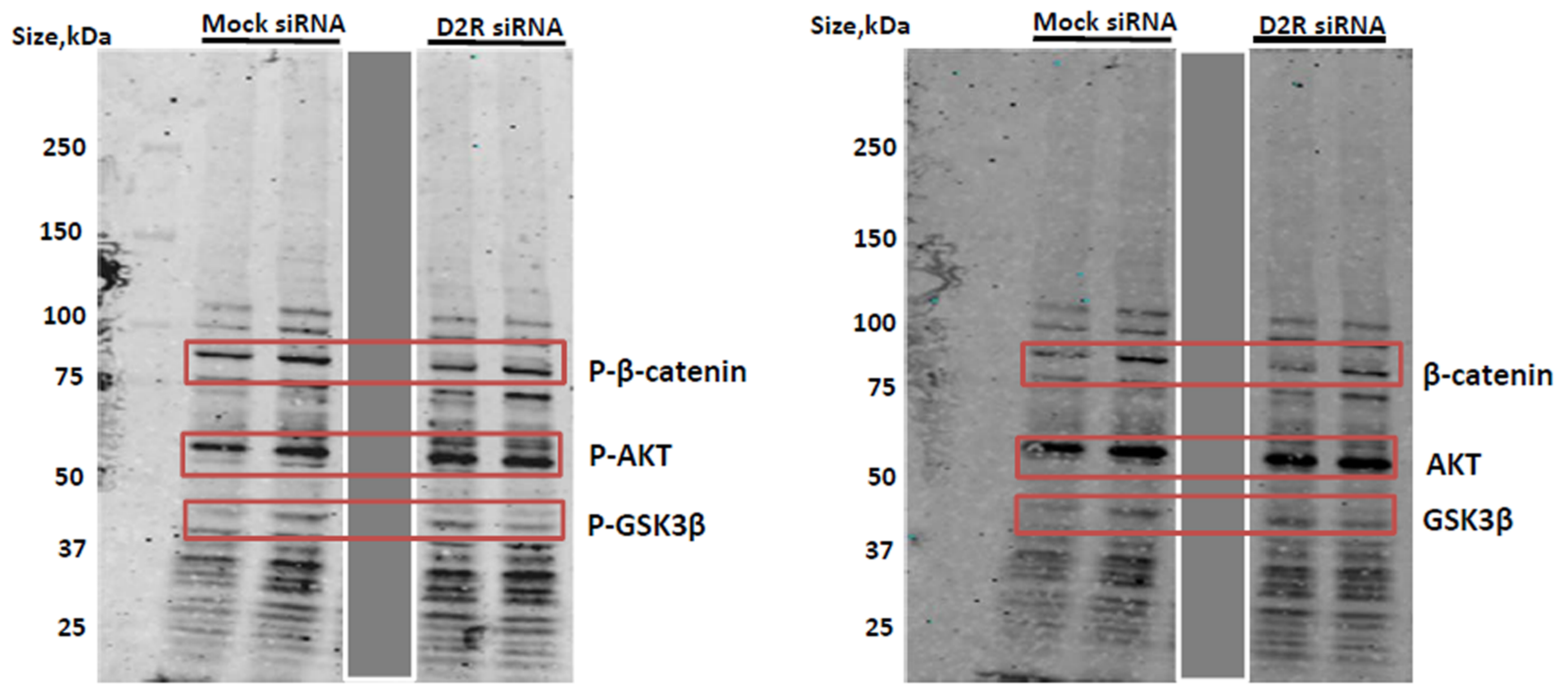

Supplementary Figure S2. Original immunoblots of phosphorylated and total β-catenin, AKT, and GSK3β protein expression.

Original immunoblots of mouse proximal tubule cells treated with D2R siRNA (20nM, 72 h) or non-silencing siRNA (Mock; 20 nM, 72 h) as described in Supplemental Figure 2. Immunoblots were first incubated with rabbit anti-phospho-β-catenin, anti-phospho-AKT and anti-phospho-GSK3β, and developed for chemiluminescence using a goat anti-rabbit secondary antibody (see Methods). The immunoblot was then washed and incubated again with rabbit anti-β-catenin, anti-AKT and anti-GSK3β and developed using a donkey antirabbit secondary antibody. These immunoblots show qualitative changes in all three phosphorylated and total forms of the respective proteins within the same sample.

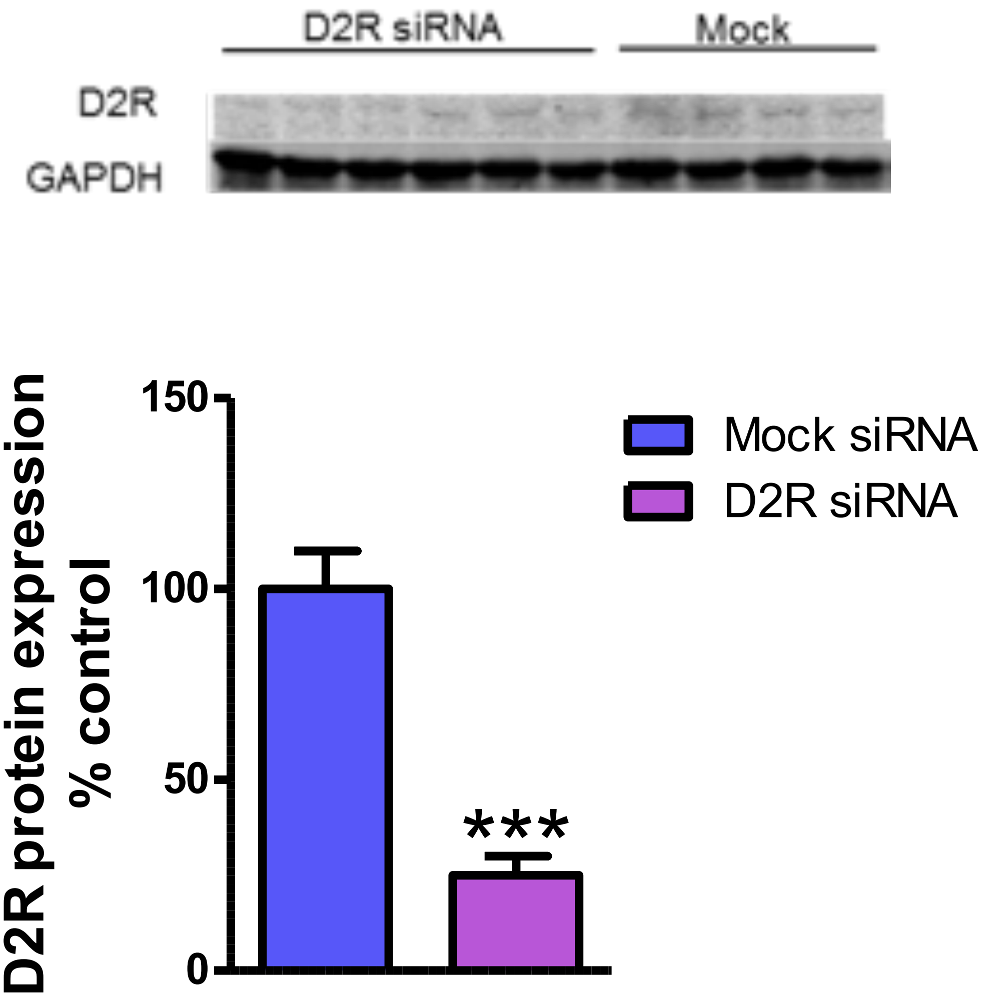

Supplementary Figure S3. D2R siRNA effectively downregulates D2R protein expression.

Quantification of D2R protein in mouse renal proximal tubule cells treated with D2R siRNA (20 nM, 72 h) or non-silencing siRNA (Mock; 20 nM, 72 h). D2R siRNA (n=6) significantly decreased levels of D2R protein by 75% relative to the non-silencing siRNA control (n=4). Representative immunoblots are shown in the inset. Results are expressed as the percentage of the control and represented as the mean ± SEM from n=4-6 independent experiments per group. ***P<0.05, Student's t-test.

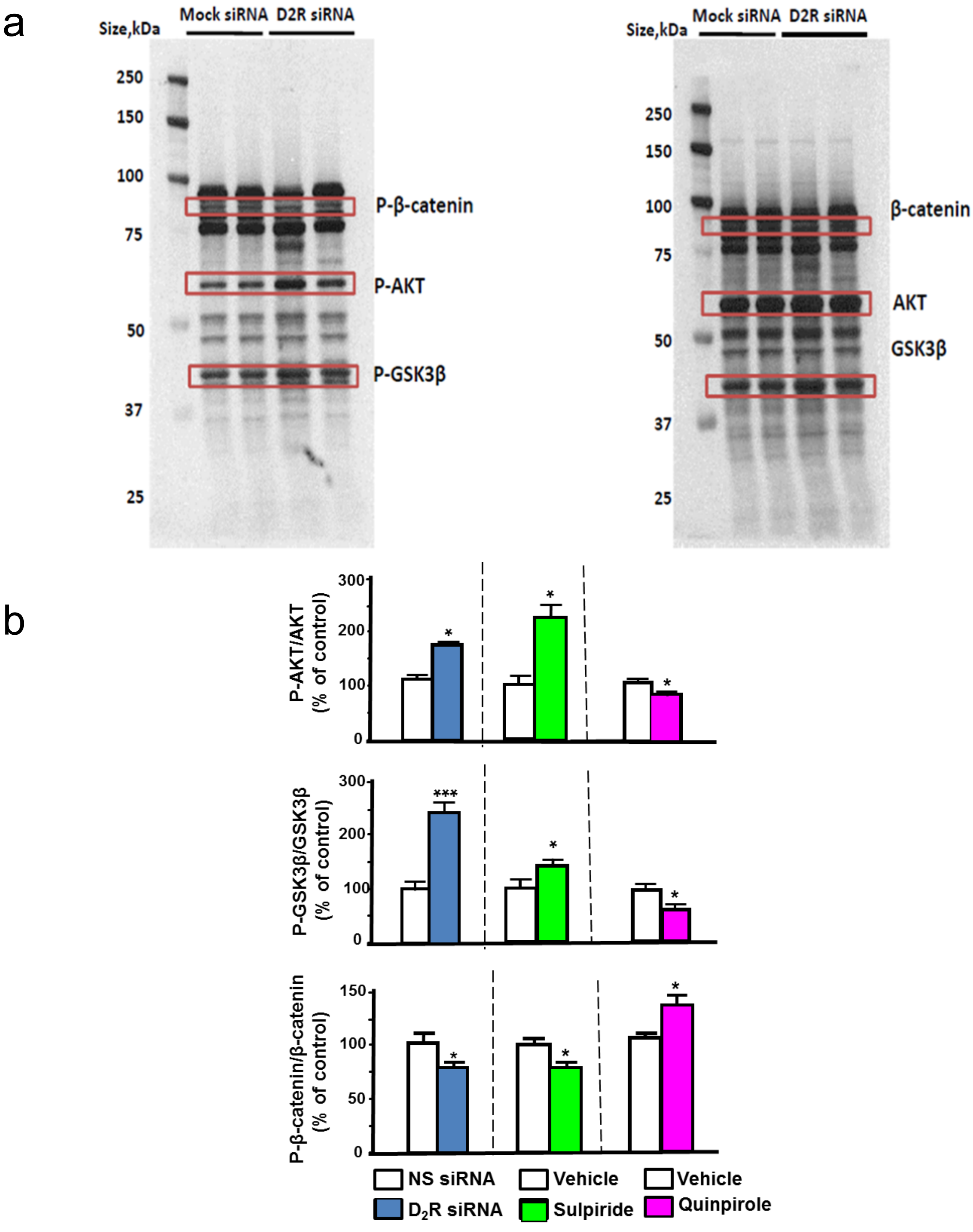

Supplementary Figure S4. Quantification of phosphorylated and total β-catenin, AKT, and GSK3β proteins in response to changes in D2R expression and function in human proximal tubules. (a) Representative immunoblots of human proximal tubule cells treated with D2R siRNA (20 nM, 72h) or non-silencing siRNA (Mock; 20 nM, 72h) as described in Supplemental Figure 2. Immunoblots were first incubated with rabbit anti-phospho-β-catenin, anti-phospho-AKT and anti-phospho-GSK3β, and developed for chemiluminescence using a goat antirabbit secondary antibody. The immunoblot was then washed and incubated again with rabbit anti-β-catenin, anti-AKT and anti-GSK3β antibodies and developed using a donkey anti-rabbit secondary antibody. These immunoblots show qualitative changes of all three phospho- and total proteins in the same sample. (b) Quantification of the immunoblots from cells treated with D2R siRNA, sulpiride and quinpirole and their respective controls (see Methods). ***P<0.001; *P<0.05, Student's t-test n=3-5/group. Data are represented as the mean ± SEM.

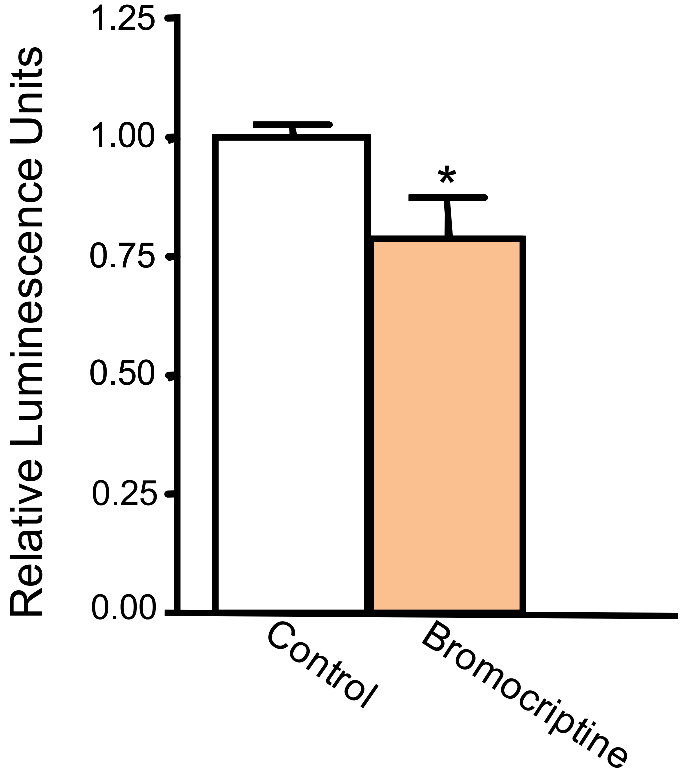

Supplementary Figure S5. Effects of D2R modulation on Wnt signaling in INS-1E cells.

Overnight treatment of rat pancreatic β-cell-derived INS-1E cells with D2R agonist bromocriptine (10 μM) significantly inhibited TCF/LEF transcriptional activity, compared with the untreated control (P=0.02). Data are normalized to untreated reporter for transfection efficiency by constitutively expressed *Renilla* luciferase. Results are represented as the mean ± SEM for two independent experiments performed in triplicate. *P<0.05, Student's t-test.

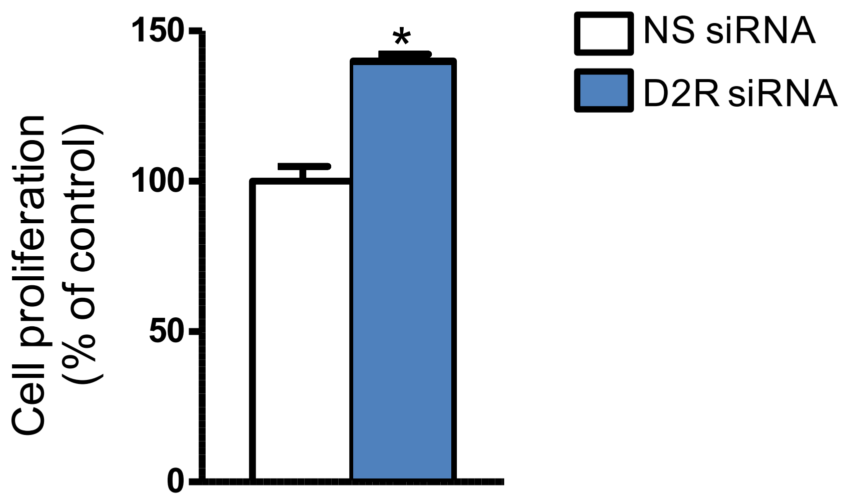

Supplementary Figure S6. D2R knockdown enhances cell proliferation in mouse renal proximal tubule cells.

Mouse renal proximal tubule cells were transfected with D2R siRNA or the non-silencing (NS) siRNA control (72 h) and assessed for cell proliferation using BrdUrd, a thymidine analog (24 h incubation). siRNA-induced D2R knockdown significantly increased cell proliferation by 41% compared with control. Results are normalized as a percentage of the NS siRNA control and represented as the mean ± SEM from 4-5 independent experiments. *P< 0.05, Student's t-test. Data are represented as the mean ± SEM.

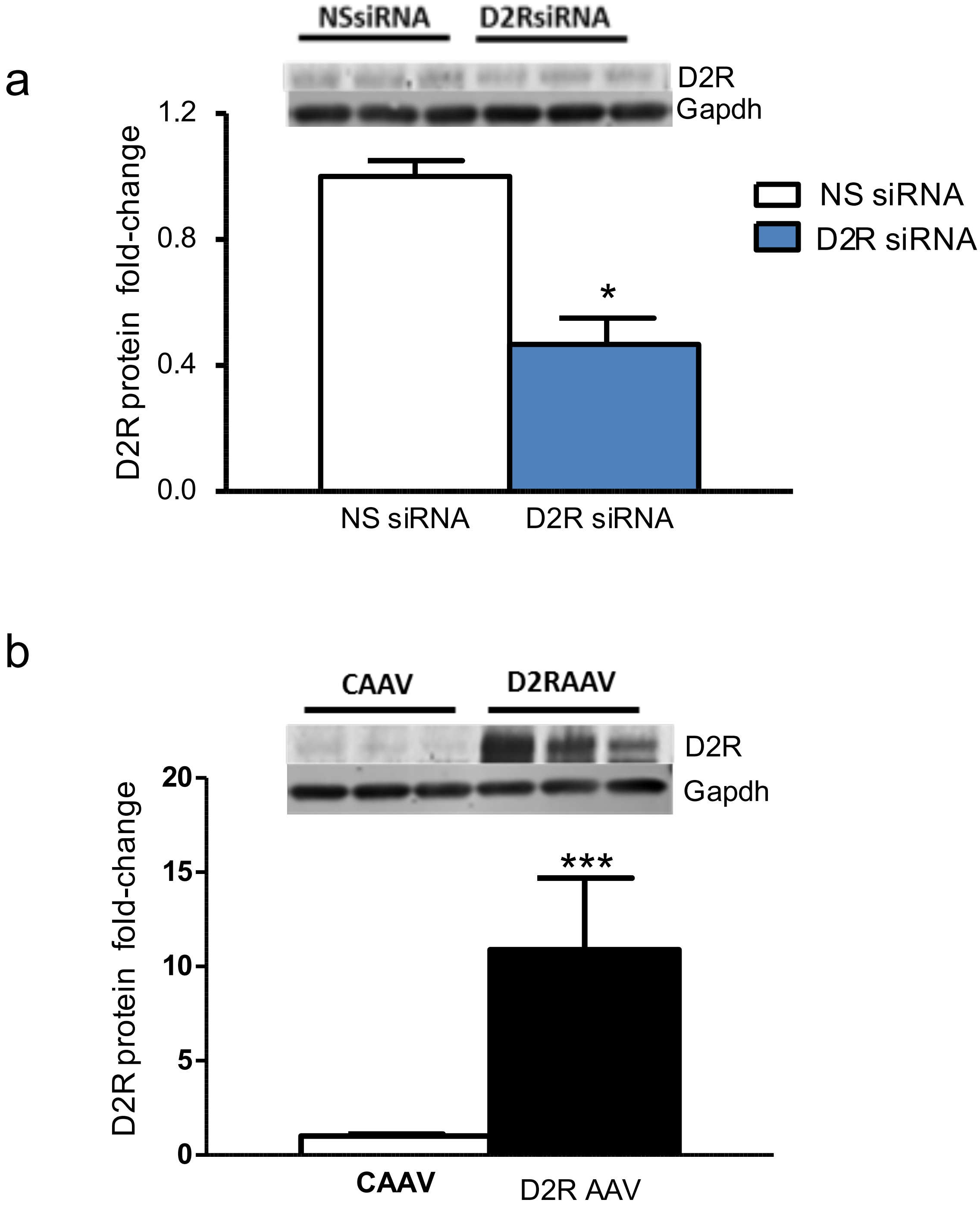

Supplementary Figure S7. Quantification of mouse renal cortex D2R protein *in vivo* in response to knockdown or overexpression.

(a) Mice underwent renal subcapsular siRNA infusion in the left kidney with either control non-silencing (NS) siRNA or D2R siRNA (3 μg/day, 28 days) via an osmotic minipump. Infusion of D2R siRNA significantly decreased the expression of D2R protein in the left renal cortex by 60% relative to the control; D2R expression was quantified by western blot with data normalized to Gapdh expression. Data are represented as the mean ± SEM. *P< 0.05, Student's t-test, n=5-6/group.

(b) D2R expression was rescued in renal cortex of mice with prior renal-selective D2R siRNA knockdown by retrograde left ureteral infusion (1×10^{11} viral genome particles) at 14 days after starting the siRNA treatment. Mice treated with D2R adeno-associated virus (D2R AAV) exhibited an ~10-fold increase in D2R protein levels (P<0.001), relative to control adeno-associated virus (CAAV). Representative immunoblots demonstrating D2R AAV and CAAV effects on D2R expression are shown in the inset. All results are represented as the mean ± SEM. ***P<0.001, Student's t-test, n=3-4/group.

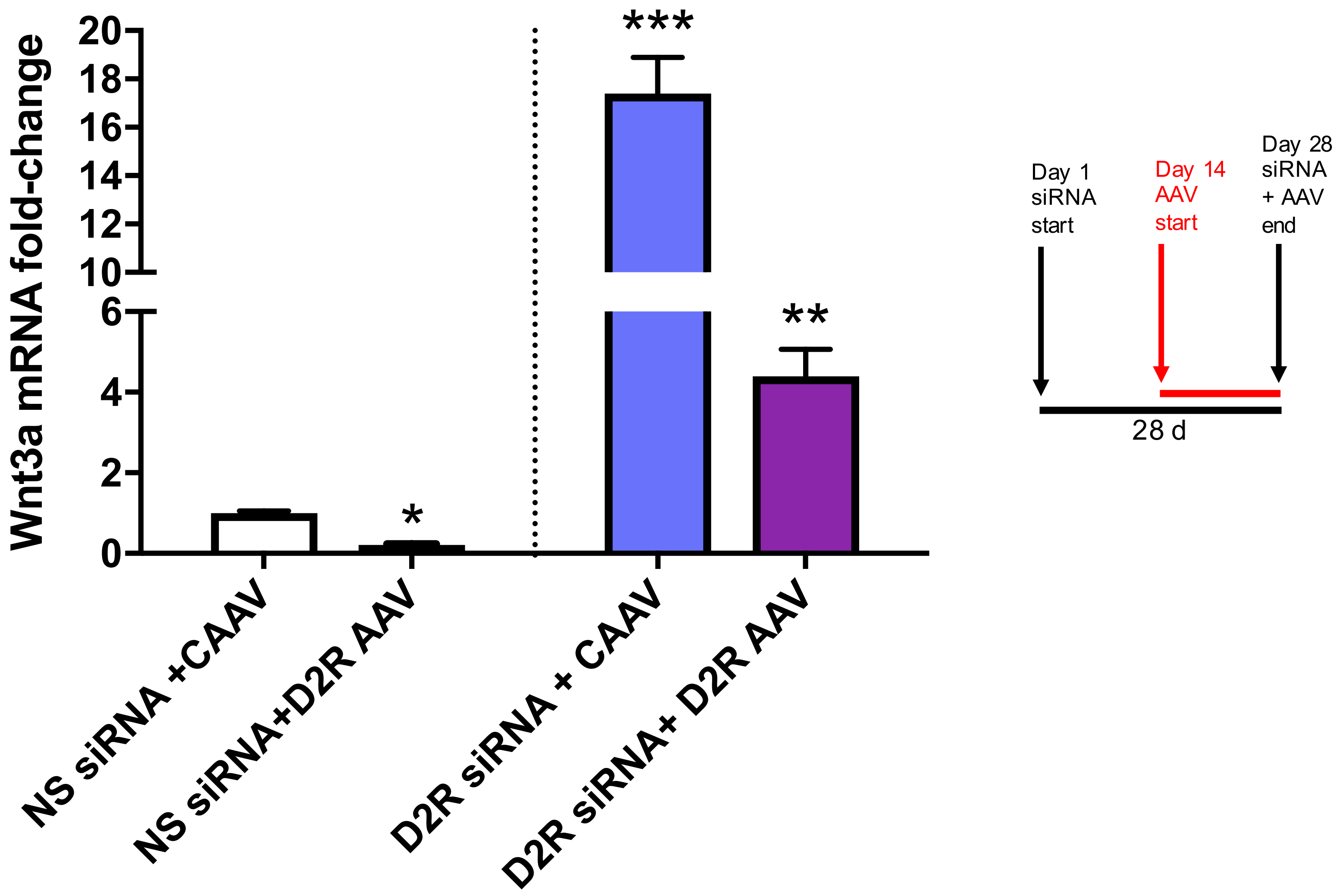

Supplementary Figure S8. D2R overexpression reduces renal Wnt3a mRNA levels *in vivo*.

Mice underwent 28-day treatment with the non-silencing siRNA control (NS siRNA) or D2R siRNA (3 µg/day siRNA) by renal subcapsular infusion of the left kidney. On day 14 of siRNA treatment, these animals received a retrograde ureteral infusion of AAV (1×10^{11} viral genome particles) either containing an empty control construct (CAAV) or the coding region of human D2R (D2R AAV) in the same left kidney. Mice were sacrificed 14 days later and Wnt3a mRNA expression in the left kidney was determined by quantitative RT-PCR; alongside is the schedule for the respective siRNA and AAV treatments. D2R overexpression by D2R AAV treatment significantly reduced Wnt3a expression in NS siRNA-treated kidney by 79%; results were normalized to the NS siRNA/CAAV control. D2R siRNA knockdown significantly increased Wnt3a expression. Concomitant D2R overexpression by D2R AAV partially reversed the increase in Wnt3a expression; results were normalized to the D2R siRNA/CAAV control. All data are represented as the mean ± SEM. *P<0.05, ** P<0.01, ***P<0.001; ANOVA followed by post-hoc Holm-Sidak analyses, n=4/group in all conditions.

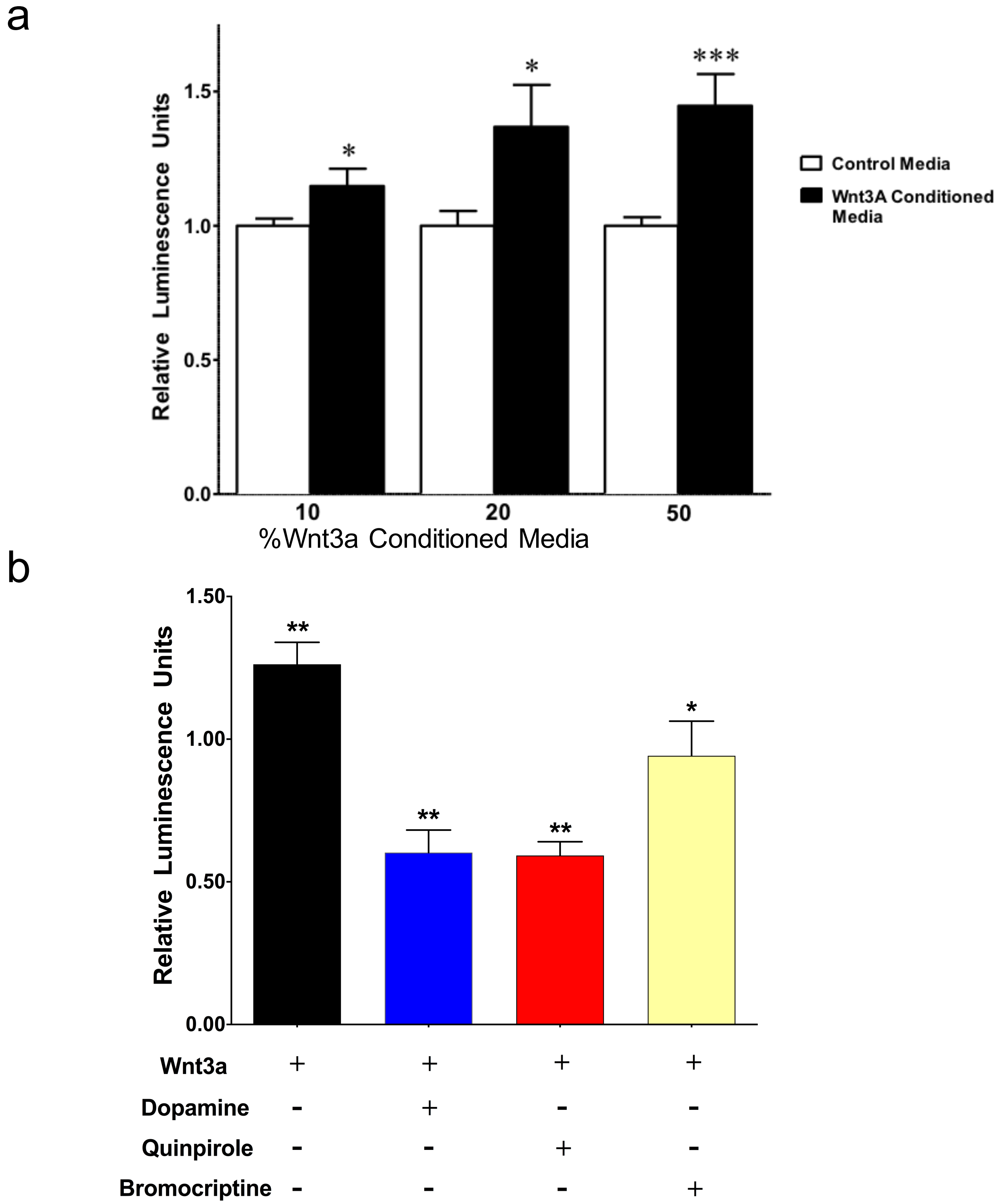

Supplementary Figure S9. D2R modulates endogenous Wnt3a-dependent signaling in INS-1E cells.

(a) INS-1E cells exhibit endogenous Wnt3a signaling. Overnight treatment (12-14 h) with increasing amounts of Wnt3a-conditioned media elicited a significant concentration-dependent increase in TCF/LEF-mediated transcription of a luminescent luciferase reporter relative to comparable amounts of Wnt3a-free control medium (10%: n=9, P=0.025; 20%: n=12, P=0.018; 50%: n=12, P=0.0007). *P<0.05, ***P<0.001; Student's t-test. (b) Co-treatment of INS-1E cells with D2R agonists: dopamine, quinpirole or bromocriptine (all drugs: 10 μM, 12-14 h) and 50% Wnt3a-conditioned medium all significantly decreased Wnt3a-stimulated TCF/LEF reporter activity (Wnt3a alone: P=0.005; Wnt3a+dopamine: P=0.003; Wnt3a+quinpirole: 0.006; Wnt3a+bromocriptine: P=0.014). All results represent the ratio of Firefly to *Renilla* luciferase activity which are normalized to the respective control media concentrations. Data are represented as the mean ± SEM for experiments performed in triplicate on 2-4 separate experimental days in all conditions. *P<0.05, **P<0.01; Student's t-test.
