# Supplementary material for: Dopamine D2 receptor modulates Wnt expression and control of cell proliferation
Source: Sci Rep. 2019 Nov 14;9:16861. doi: 10.1038/s41598-019-52528-4 (PMC6856370; doi:10.1038/s41598-019-52528-4)
Supplement: Supplementary file 2 — Dataset 1 [file 41598_2019_52528_MOESM2_ESM.pdf]

Supplementary Information File

Dopamine D_2 receptor modulates Wnt expression and control of cell proliferation

Fei Han[1%], Prasad Konkalmatt[1%], Chaitanya Mokashi[2], Megha Kumar[1], Yanrong Zhang[1], Allen Ko[3], Zachary J. Farino[4], Laureano D. Asico[1], Gaosi Xu[1], John Gildea[5], Xiaoxu Zheng[1], Robin A. Felder[5], Robin E.C. Lee[2], Pedro A. Jose[1,6], Zachary Freyberg[4,7,§*], Ines Armando[1§*]

From the [1]Department of Medicine, School of Medicine and Health Sciences, The George Washington University, Washington DC, 20052; [2]Department of Computational & Systems Biology, University of Pittsburgh, Pittsburgh, PA 15213; [3]Institute of Human Nutrition, College of Physicians & Surgeons, Columbia University, New York, NY 10032; [4]Department of Psychiatry, University of Pittsburgh, Pittsburgh, PA 15213; [5]Department of Pathology, The University of Virginia, Charlottesville, VA 22904; [6]Department of Pharmacology and Physiology, School of Medicine and Health Sciences, The George Washington University, Washington DC, 20052; [7]Department of Cell Biology, University of Pittsburgh, Pittsburgh, PA 15213

[%]Authors contributed equally

[§]Co-corresponding authors

* Corresponding Authors:

Zachary Freyberg, MD, PhD
University of Pittsburgh
3811 O'Hara Street
BST, W1640
Pittsburgh, PA 15213
Tel: 646-595-8317
freyberg@pitt.edu

Ines Armando, PhD
The George Washington University
2300 Eye Street
Ross Hall Suite 738
Washington, D.C. 20037
Tel: 202-994-0159
iarmando@gwu.edu

List of included materials: **Supplementary Dataset 1**

Figure 1b

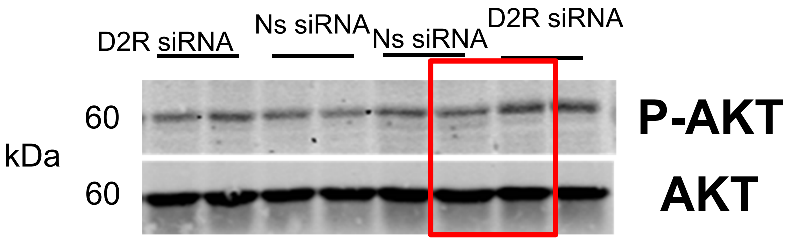

D2R siRNA
Ns siRNA
Ns siRNA
D2R siRNA
60
kDa
60
P-AKT
AKT

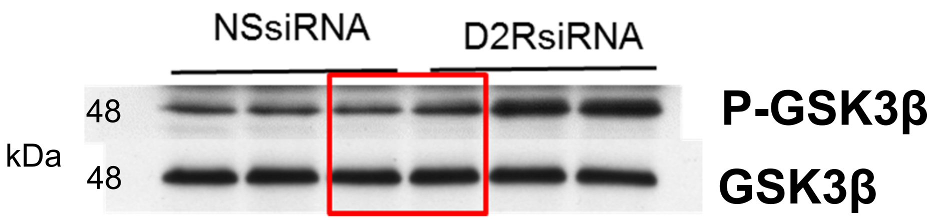

NSsiRNA
D2RsiRNA
48
kDa
48
P-GSK3β
GSK3β

Figure 1b

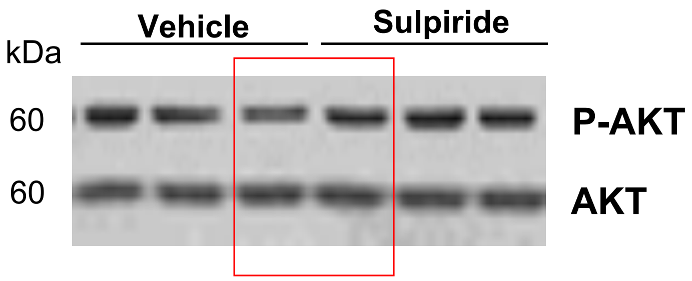

Vehicle
Sulpiride
kDa
60
60
P-AKT
AKT

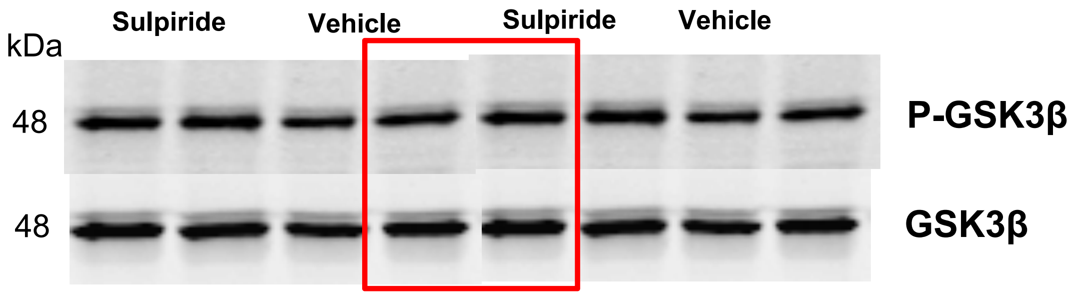

Sulpiride
Vehicle
Sulpiride
Vehicle
kDa
48
48
P-GSK3β
GSK3β

Figure 1b

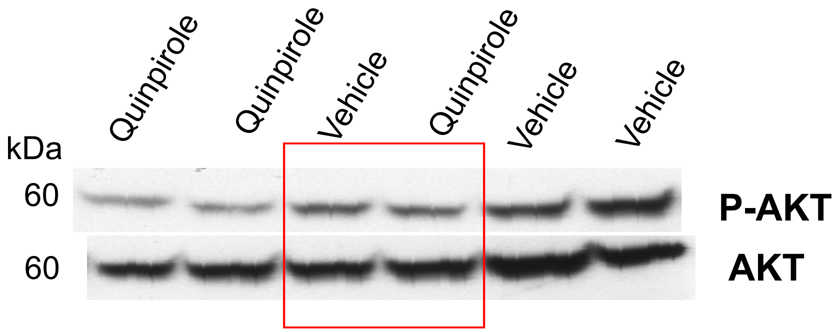
Quinpirole
Quinpirole
Vehicle
Quinpirole
Vehicle
Vehicle
kDa
60
60
P-AKT
AKT

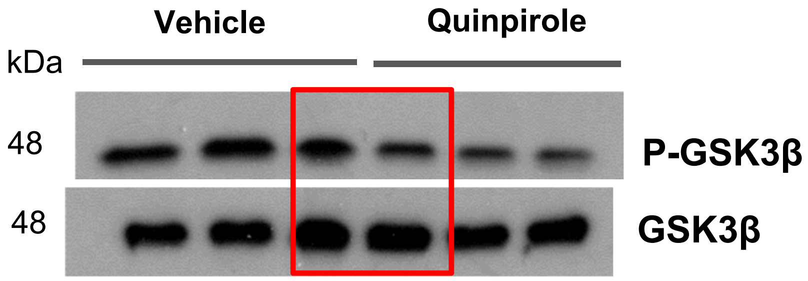
Vehicle
Quinpirole
kDa
48
48
P-GSK3β
GSK3β

Figure 2a

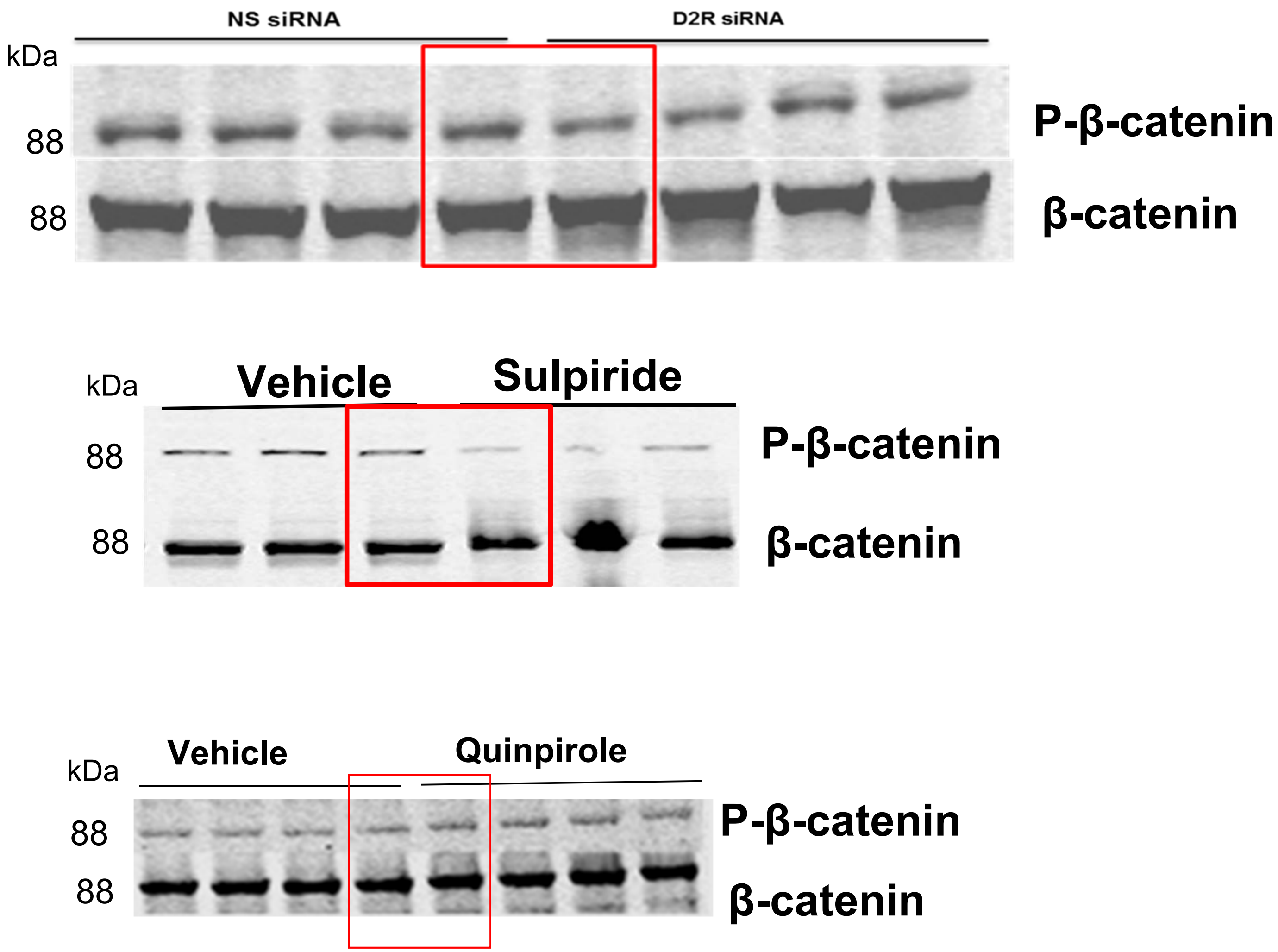

NS siRNA
D2R siRNA
kDa
88
88
P-β-catenin
β-catenin
kDa
Vehicle
Sulpiride
88
88
P-β-catenin
β-catenin
kDa
Vehicle
Quinpirole
88
88
P-β-catenin
β-catenin

Figure 3b

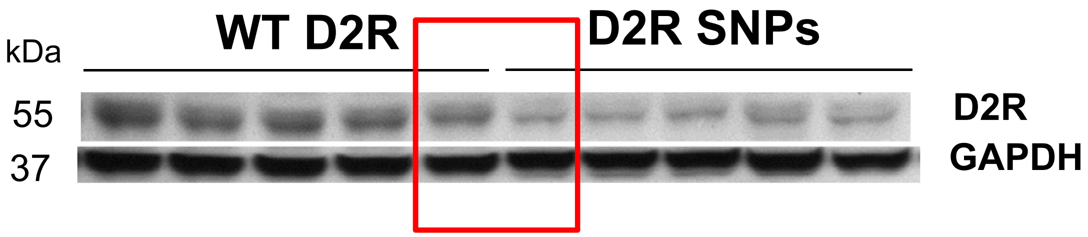

WT D2R
D2R SNPs
kDa
55
37
D2R
GAPDH

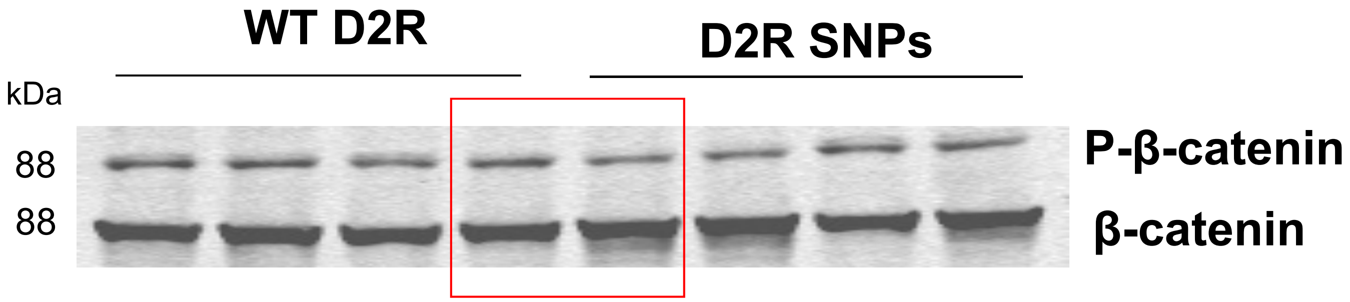

WT D2R
D2R SNPs
kDa
88
88
P-β-catenin
β-catenin

Figure 3c

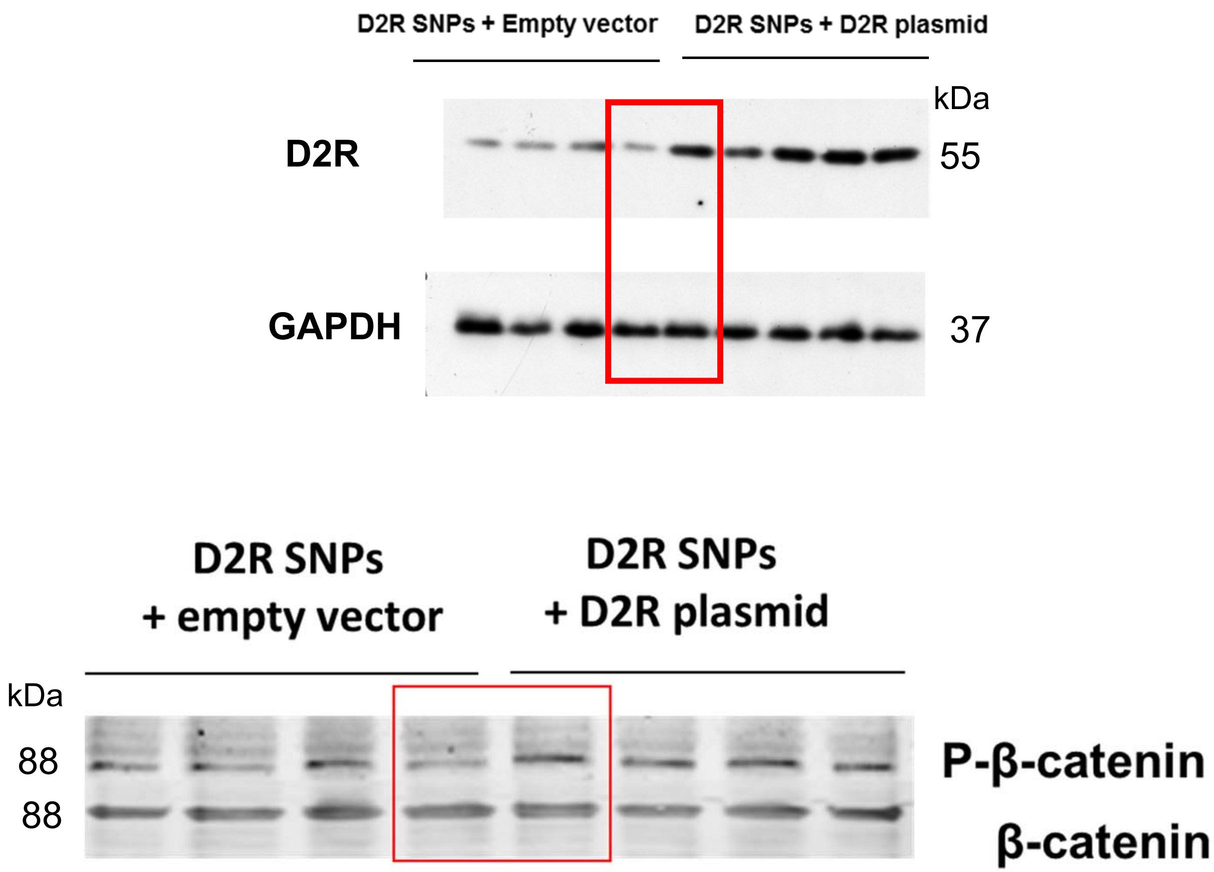

D2R SNPs + Empty vector
D2R SNPs + D2R plasmid
kDa
D2R
55
GAPDH
37
D2R SNPs
+ empty vector
D2R SNPs
+ D2R plasmid
kDa
88
88
P-β-catenin
β-catenin

Figure 4a

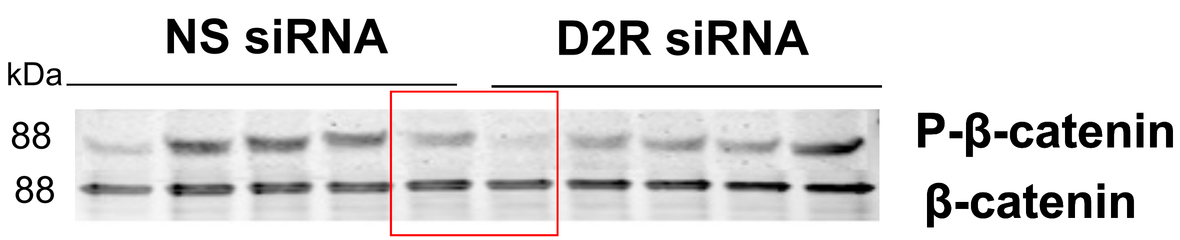

Figure 4c

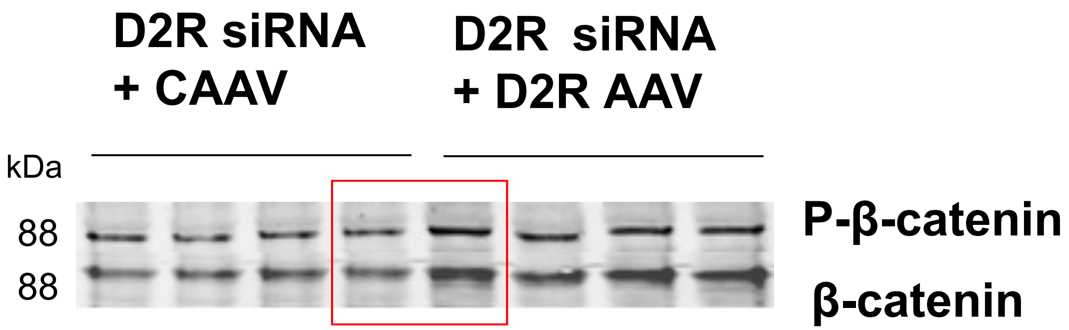

Figure 5c

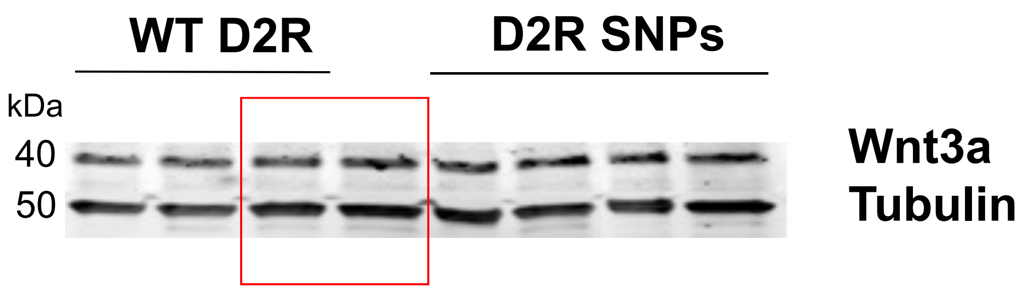

Figure 5e

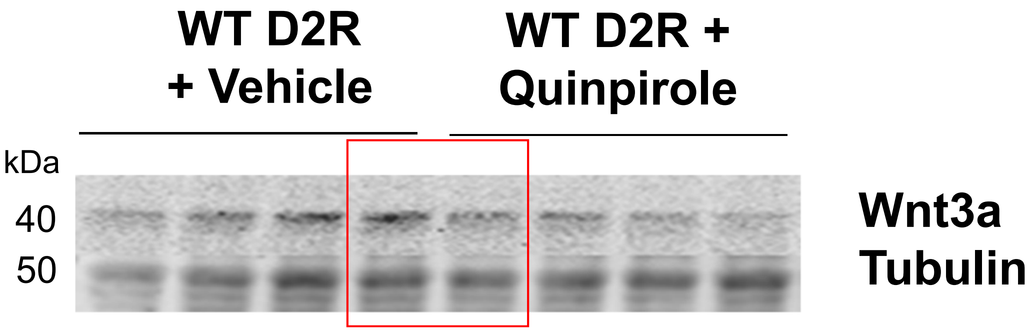

Figure 5d

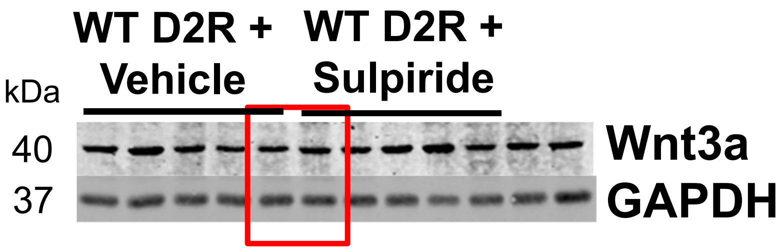

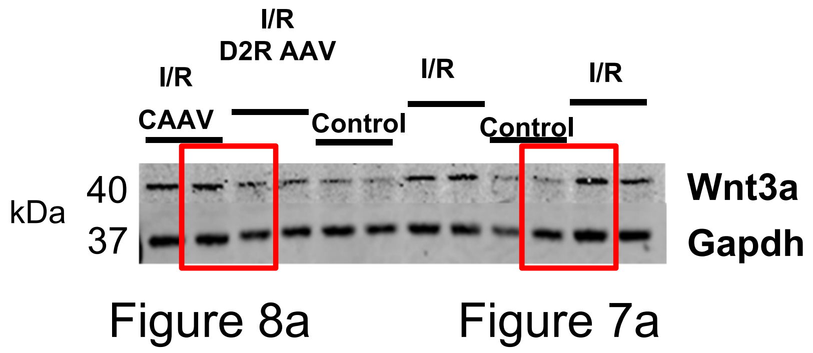
I/R
CAAV
I/R
D2R AAV
Control
I/R
Control
I/R
kDa
40
37
Wnt3a
Gapdh
Figure 8a
Figure 7a
